# Direct Identification and Quantification of Flavonoids and Their Structural Isomers Using Ambient Ionization Tandem Mass Spectrometry

**DOI:** 10.1002/rcm.10169

**Published:** 2025-11-08

**Authors:** Yanqiu Wang, Liping Xu, Tiange Gu, Hongli Li, David Da Yong Chen

**Affiliations:** ^1^ Jiangsu Collaborative Innovation Center of Biomedical Functional Materials, Jiangsu Key Laboratory of Biomedical Materials, School of Chemistry and Materials Science Nanjing Normal University Nanjing China; ^2^ Department of Chemistry University of British Columbia Vancouver British Columbia Canada; ^3^ State Key Laboratory of Analytical Chemistry for Life Science, School of Chemistry and Chemical Engineering Nanjing University Nanjing China

**Keywords:** computer program, direct analysis in real time ionization, flavonoid aglycone subtype, flavonoid isomers, tandem mass spectrometry

## Abstract

**Rationale:**

Flavonoids are phenolic compounds with many health‐benefiting properties. However, differentiating different types of flavonoids and their isomers is challenging due to their highly similar structures of various subtypes and different numbers and sites of substituents. Timely quality evaluation of flavonoid‐based products is currently almost impossible.

**Methods:**

An ambient ionization method of direct analysis in real time (DART) ion source and tandem mass spectrometry (MS) was used to characterize the fine structures of flavonoids. Different flavonoid subtypes and their isomers with varied numbers and sites of substituents were subjected to DART ionization and collision‐induced fragmentation MS analysis.

**Results:**

Seven classes of flavonoids, including methoxy‐substituted compounds, exhibited distinctive fragmentation pathways such as retro‐Diels‐Alder reactions, cross‐ring cleavages, and neutral losses. Many flavonoid isomers produced diagnostic MS^2^ and MS^3^ fragments through DART‐tandem MS, enabling direct identification of various isomers within mixtures. An identification workflow was developed, culminating in the creation of a computational tool called FlavoFinder, which automatically determines flavonoid aglycone subtypes and their isomeric structures.

**Conclusions:**

The method and the structural elucidation program were successfully used for the qualitative and quantitative analysis of different flavonoid isomers from real samples. The analysis procedure is high‐throughput and is capable of characterizing complex flavonoid structures without extensive sample pretreatment and front‐end chromatographic separations.

## 1 Introduction

Flavonoids are natural products composed of aglycone skeletons and their derivatives, with anti‐oxidative, anti‐inflammatory, anti‐mutagenic, and anti‐carcinogenic properties, and can modulate key cellular enzyme functions [1, 2, 3, 4, 5, 6]. Flavonoid aglycones have different subtypes, and each subtype can be connected with different numbers of substituents such as hydroxyl and methoxy moieties at different positions, with a large number of structural isomers that may have different functions in biological systems [7, 8, 9]. Methods that can fully characterize flavonoids' fine structures, including subclasses and isomeric features, are needed before the functional characterization of these compounds can be achieved.

Multistage tandem mass spectrometry can elucidate chemical structures by means of consecutively selecting product ions from the previous collision products to the n^th^ order (MS^n^) [10, 11]. Some reports demonstrated the use of fragmentation characteristics of flavonoids from electrospray ionization (ESI) and collision‐induced dissociation (CID) for their structure determination [12, 13, 14]. Identification and quantification of flavonoid isomers in real samples remain difficult, because the product ion patterns from these compounds are very similar, especially for flavonoids with only minor structural differences [15]. Due to the number of flavonoid substances that are present in real samples, liquid chromatography (LC) is often needed to separate the different components before the ESI‐MS^n^ step [16, 17, 18, 19, 20]. A full collision energy (ce) ramp‐MS^2^ analysis including MS^2^ spectra at any ce and optimal ce showed great potential in structural profiling of flavonoids. However, this method involves the acquisition of a large amount of data for processing and calculations [21]. Zhang et al. developed a workflow to annotate plant aglycones and glycosides using untargeted LC–MS^2^ [22], where information from different databases and literature were used to build libraries. An LC–MS^2^ strategy for flavonoid screening and identification was developed by combining mass defect filtering and diagnostic product ion analysis [23]. However, LC–MS techniques have limitations including long separation times and tedious method development procedures. LC methods developed for characterization of a limited number of isomeric flavonoids required special columns and methods. The lack of robustness and transferability of the methods limits the wide spread adoption of these methods [24, 25].

In addition to liquid chromatography, gas‐phase separation techniques such as ion mobility‐mass spectrometry (IM‐MS) have been applied to characterize certain flavonoid structural isomers based on their collision cross sections [26, 27, 28, 29]. However, ion mobility spectrometry has limited capability in separating large or diverse groups of isomeric flavonoids. Furthermore, some computational tools have been developed to analyze flavonoid structures by integrating multiple types of data, including retention time, mass spectral dissociation patterns, ultraviolet (UV) absorption, and/or nuclear magnetic resonance (NMR) data [30, 31]. Nevertheless, integrating these disparate tools and the need to reproduce specific experimental conditions can be prohibitively complex for most users in practice. Characterization of flavonoid structures including aglycone subtypes and isomers from complex samples without front‐end liquid or gas‐phase chromatographic separation would be a significant improvement over the currently available methods.

Direct analysis in real time (DART) is one of the ambient ionization techniques for MS [32, 33]. It is simple, fast, and capable of high‐throughput analysis in an open environment with minimal sample pretreatment [34, 35]. Excited state atoms of inert gases (helium or nitrogen) interact with analytes in the sample and undergo Penning‐type processes to generate analyte ions. DART‐MS has been used in different applications, especially for the rapid analysis of pesticides, illicit drugs, contaminants, and active ingredients in natural products and pharmaceuticals [36, 37]. However, due to the elimination of the preceding separation techniques, its use for the structural elucidation of bioactive substances, including isomer characterization, is very limited [38, 39]. We have reported the successful identification of carbohydrate and ginsenoside isomers using DART‐tandem MS [40, 41]. However, not much on the characterization of flavonoid structures using DART‐MS has been reported.

In this work, we report a simple and robust method based on DART‐tandem MS features to directly characterize flavonoid structures, including aglycone subtypes and isomers, even when they are present in mixtures. A publicly accessible program, FlavoFinder, was created to perform the structural annotation of flavonoids in real‐life samples. The FlavoFinder program is capable of batch processing, and is designed for non‐experts in the field of structural determination.

## 2 Materials and Methods

### 2.1 Chemicals and Materials

Some of the commonly available flavonoids were used to establish the database used for structural identification. Flavonoids of chrysin, liquiritigenin, pinocembrin, genistein, galangin, naringenin, phloretin, wogonin, biochanin A, luteolin, kaempferol, fisetin, catechin, diosmetin, morin, hesperetin, gallocatechin, epigallocatechin, and myricetin were purchased from Tongtian Industrial (Shanghai, China). Daidzein, apigenin, baicalein, and quercetin were purchased from Macklin Industrial (Shanghai, China). Isoliquiritigenin, butein, and 4‐methoxychalcone were purchased from Yuanye Industrial (Shanghai, China). Methanol, acetone, petroleum ether, ethyl acetate, and n‐butanol were obtained from Sinopharm Chemical Reagent (Shanghai, China). Acetyl‐rimantadine was purchased from Haohong Biomedical Technology Company (Shanghai, China). Wahaha purified water (WaHaha Ltd., Nanjing, China) was used. Real samples including cajan leaves and rooibos tea were purchased from online stores in China. Ginkgo leaves were collected from the Xianlin campus of Nanjing Normal University (Nanjing, China).

### 2.2 Sample Preparation

All flavonoid standards were dissolved in methanol with a concentration of 1.0 mg mL^−1^ as the stock solutions. The stock solutions were diluted with methanol to make a series of working solutions with concentrations of 0.1, 0.5, 1, 5, 10, 50, 100, and 200 μg mL^−1^. Internal standard (IS) of acetylated rimantadine was prepared at a concentration of 0.1 mg mL^−1^ in methanol, and the IS of 1 μL was spiked into different flavonoid working solutions. A mixture containing five groups of flavonoid isomers, namely, (1) daidzein and chrysin, (2) liquiritigenin and pinocembrin, (3) apigenin, genistein, baicalein and galangin, (4) wogonin, biochanin A, and calycosin, and (5) luteolin, kaempferol, and fisetin, was prepared. The concentrations were 0.2, 1.2, 0.4, 0.6, and 0.8 mg mL^−1^ for each isomeric group, respectively.

### 2.3 Preparation of Real Samples

The leaf samples were dried and smashed using a pulverizer (Tianjin AISITE Inc., China). A standard 80 mesh test sieve (Xiongchen Inc., Nanjing, China) was used to obtain the fine powder. Mechanochemical extraction (MCE) was used to extract flavonoids from the sample powders. Briefly, 75 mg of sample powder and 1.0 mL of acetone/water (v/v 7/3) were placed into a 2‐mL Lysing Matrix D tubes containing 1.4‐mm ceramic spheres. The extraction was performed with a FastPrep‐24 instrument (MP Biomedicals LLC, USA) at a speed of 4.5 m/s for 60 s. The crude extract was centrifuged (Centrifuge H‐1650, XiangYi Centrifuge Co., China) at 8000 rpm for 5 min. The supernatant was collected, and acetone was removed using a rotary vacuum evaporator (Shanghai Yarong Biochemical Instrument Factory) at 45°C. The residual water phase was extracted sequentially with petroleum ether (2 **×** 2 mL), ether (2 **×** 2 mL), ethyl acetate (2 **×** 2 mL), and n‐butanol (2 **×** 2 mL). The n‐butanol organic solvent phase was collected and evaporated to dryness at 60°C. The dried extract was re‐dissolved in 1 mL of methanol, and then the solution was spiked with IS.

### 2.4 DART‐Tandem MS Analysis

The DART‐SVP ion source (Ion Sense, Saugus, MA, USA) was connected to a high‐resolution Tribrid Orbitrap Fusion Lumos mass spectrometer (Thermo Fisher Scientific, Waltham, MA, USA) through a Vapur interface (Ion Sense, Saugus, MA, USA). High‐purity helium (99.999%) was used as the operating gas for DART ionization, and nitrogen (99.999% purity) was used in standby mode. Liquid samples of 1‐μL aliquots were pipetted onto the bottom of the DIP‐it tip and then introduced by a 12 DIP‐it holder rack on a moving rail at a speed of 0.3 mm s^−1^. All samples were analyzed in positive ion mode, and the MS system was controlled by Orbitrap Fusion Lumos 2.0 Tune (Thermo Fisher Scientific, MA, USA) software. The resolution of the Orbitrap analyzer was set to 60 000, and the temperature of the ion transfer tube was at 350°C. The maximum injection time used was 100 ms with an automatic gain control target of 2 × 10^5^. CID was used to fragment flavonoid precursor ions, and the CID energy was manually adjusted from 30 to 42 eV in this work. The activation Q was 0.25, and the isolation window was set at 1 *m/z* through a quadrupole selection. Multiple measurements (*n* ≥ 6) were conducted for each sample.

### 2.5 Data Analysis

The schematics were drawn using Microsoft Office PowerPoint 16.0 (Redmond, WA, USA). The chemical structures were drawn with ChemBioDraw Ultra 7.0 (cambridgesoft.com, MA, USA). The figures were plotted with OriginPro 2016 software (OriginLab, Northampton, MA, USA) using MS and tandem MS data exported from Xcalibur 2.1 software (Thermo Fisher Scientific, MA, USA).

### 2.6 Computer Program FlavoFinder

FlavoFinder was built using a Server Side Includes (SSI) application program. MS and tandem MS ion lists from tandem MS analysis can be imported into the program, and automatic data analysis can be performed to predict the flavonoid subtype and isomer, which can generate the report immediately. FlavoFinder is free of charge on a web site http://www.flavofinder.com, and it is easily accessible and user‐friendly for researchers. The user manual with step‐by‐step instructions is available upon request.

## 3 Results and Discussion

### 3.1 Flavonoid Aglycone Subtypes

**FIGURE 1:**
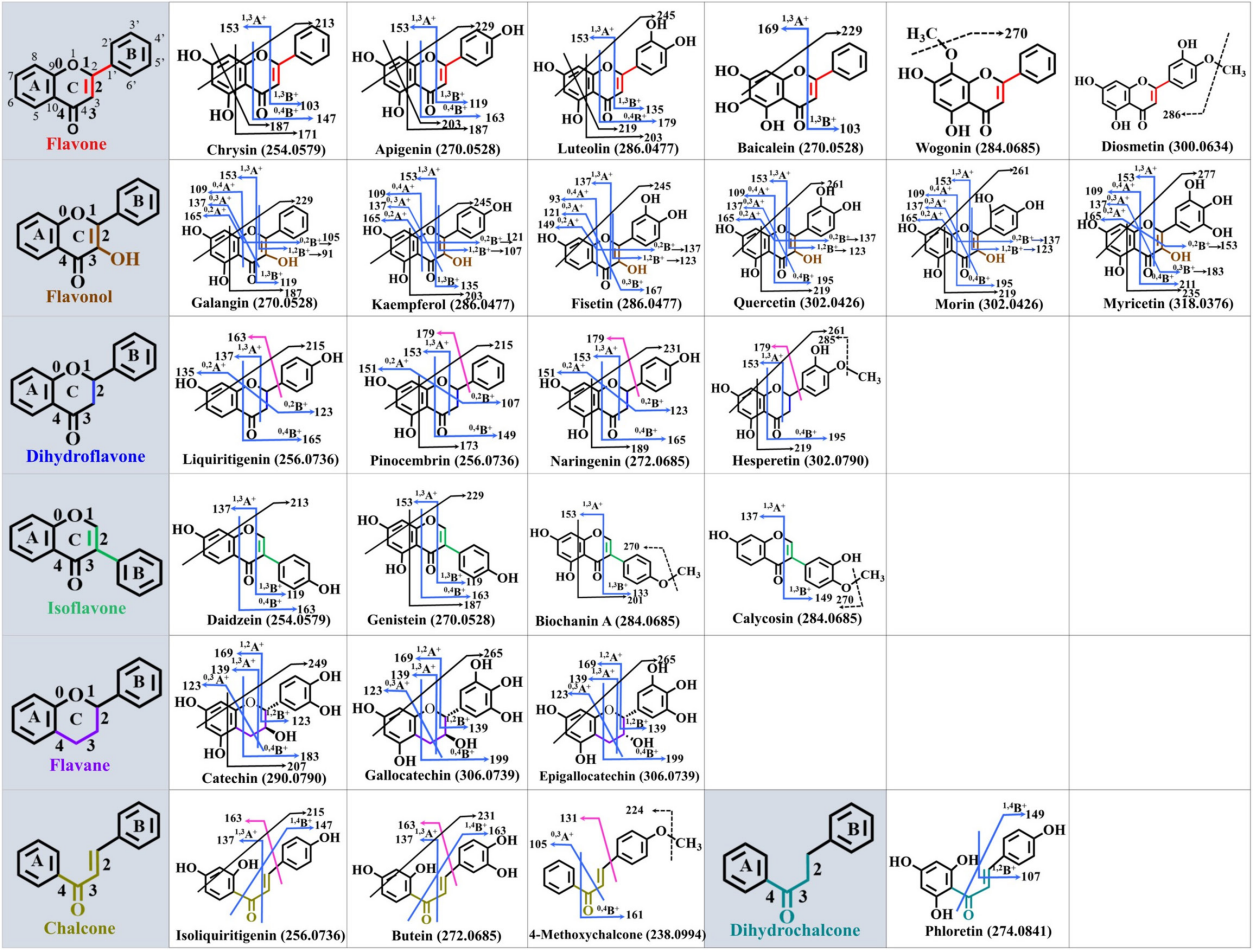
Proposed DART‐MS^2^ fragmentation pathways for different subtypes of flavonoids including flavone, flavonol, dihydroflavone, isoflavone, flavane, chalcone and dihydrochalcone. On the structures, solid black arrows indicate cross‐ring cleavages, blue arrows indicate RDA reactions, pink arrows indicate B‐ring loss, and dashed black arrows indicate methyl group loss. All the ions are protonated ions.

As shown in Figure 1, flavonoid aglycones can be divided into different subtypes, including flavones, flavonols, isoflavones, dihydroflavones, flavanes, chalcones, and dihydrochalcones. Flavonoid aglycones are often substituted with hydroxyl and methoxy groups, and owing to the different substituent type, position, and number, each type contains a subgroup of compounds. A total of 27 flavonoid standards, along with their structural characteristics, are provided in Table S1. With DART ionization, molecular ions were obtained for the tested flavonoids in full mass scan (MS^1^), and MS^2^ analysis was then performed for the [M + H]^+^ ions using CID. Retro‐Diels‐Alder (RDA) reaction, which cleaves a six‐membered cycloalkene compound to form a dienophile and an ethylene molecule [42], is widely reported in the fragmentation of flavonoids.

Figure 1 shows the RDA reactions that happened on the C ring, cross‐ring cleavages of the A ring, and the loss of the B ring for each flavonoid species. The flavone subtype of chrysin, apigenin, and luteolin exhibited similar fragmentation pathways, which were dominated by RDA reactions of ^1,3^A^+^, ^1,3^B^+^, and ^0,4^B^+^ and cross‐ring cleavages of C_2_H_2_O, C_4_H_4_O_2_, and C_3_O_2_. However, baicalein only produced ^1,3^A^+^ and ^1,3^B^+^ ions and underwent C_2_H_2_O cleavage. This is probably due to the additional –OH group on C6 of the A ring providing steric hindrance for some of the fragmentation. Flavonol generated abundant RDA reaction products, and fragments of ^1,3^A^+^, ^0,4^A^+^, ^0,3^A^+^, ^0,2^A^+^, and ^0,2^B^+^ were common for all the compounds. In addition, the –OH groups on the B ring affected RDA reactions for the generation of B^+^ ions. For example, quercetin and morin having 2 –OH groups substituted on the B ring generated ^1,2^B^+^ and ^0,4^B^+^ product ions, and myricetin with 3 –OH groups on the B ring gave ^0,3^B^+^ and ^0,4^B^+^ fragments. The studied dihydroflavone compounds had the same fragmentation patterns, giving RDA fragment ions of ^1,3^A^+^, ^0,2^A^+^, ^0,2^B^+^, and ^0,4^B^+^ and cross‐ring cleavages of C_2_H_2_O and C_4_H_4_O_2_. Moreover, the loss of the B ring was clearly observed for dihydroflavone flavonoids. Isoflavones of daidzein and genistein both generated simple RDA reaction product ions of ^1,3^A^+^, ^1,3^B^+^, and ^0,4^B^+^ on the C ring and showed cross‐ring cleavage of C_2_H_2_O and C_4_H_4_O_2_ on the A ring as well. RDA fragments of ^1,2^A^+^, ^1,3^A^+^, ^0,3^A^+^, ^1,2^B^+,^ and ^0,4^B^+^ were found for all the studied flavane compounds including catechin, gallocatechin (GC), and epigallocatechin (EGC). The cleavages of C_2_H_2_O and C_4_H_4_O_2_ were observed for catechin which has two –OH groups (C4′, C5′) on the B ring. However, the cleavage of only C_2_H_2_O occurred for GC and EGC, and they have three –OH groups (C3′, C4′, C5′) on the B ring. Different from other flavonoid subtypes above, the C rings of chalcone and dihydrochalcone are open. Chalcone flavonoids of isoliquiritigenin and butein both generated RDA fragments of ^1,3^A^+^ and ^1,4^B^+^ and cross‐ring cleavage of C_2_H_2_O. In addition, the loss of the B ring was observed for chalcone compounds as well. Phloretin belongs to the dihydrochalcone type, and it only generated RDA reaction products of ^1,2^B^+^ and ^1,4^B^+^, without cross‐ring cleavages observed on the A ring. More numbers of dihydrochalcone flavonoids need to be investigated for validation of their fragmentation features.

For methoxy‐substituted flavonoid compounds including wogonin, diosmetin, hesperetin, biochanin A, calycosin, and 4‐methoxychalcone, they all underwent significant CH_3_· elimination. The RDA reactions of these compounds can be either fully or partially inhibited. For example, no RDA reactions were observed for methoxy‐substituted flavones including wogonin and diosmetin. RDA products of ^0,2^A^+^ and ^0,2^B^+^ were not produced for hesperetin, and no ^0,4^B^+^ fragment was observed for methylated isoflavones of biochanin A and calycosin. Flavonoid of 4‐methoxychalcone generated RDA ions of ^0,3^A^+^ and ^0,4^B^+^, which are different from non‐methylated chalcones. This could be caused by the attached methoxy group or be affected by the different A ring structure. Moreover, the methoxy substituent can also affect the cross‐ring cleavages on the A ring of flavonoids. For example, the cross‐ring cleavages were completely inhibited for wogonin and diosmetin of flavone type and partially inhibited for biochanin A and calycosin of isoflavone type. Notably, the neutral losses of H_2_O and CO as well as cleavage of CHO are also common for MS^2^ cleavage of flavonoid aglycones of different types, which are not annotated in the structures.

**FIGURE 2:**
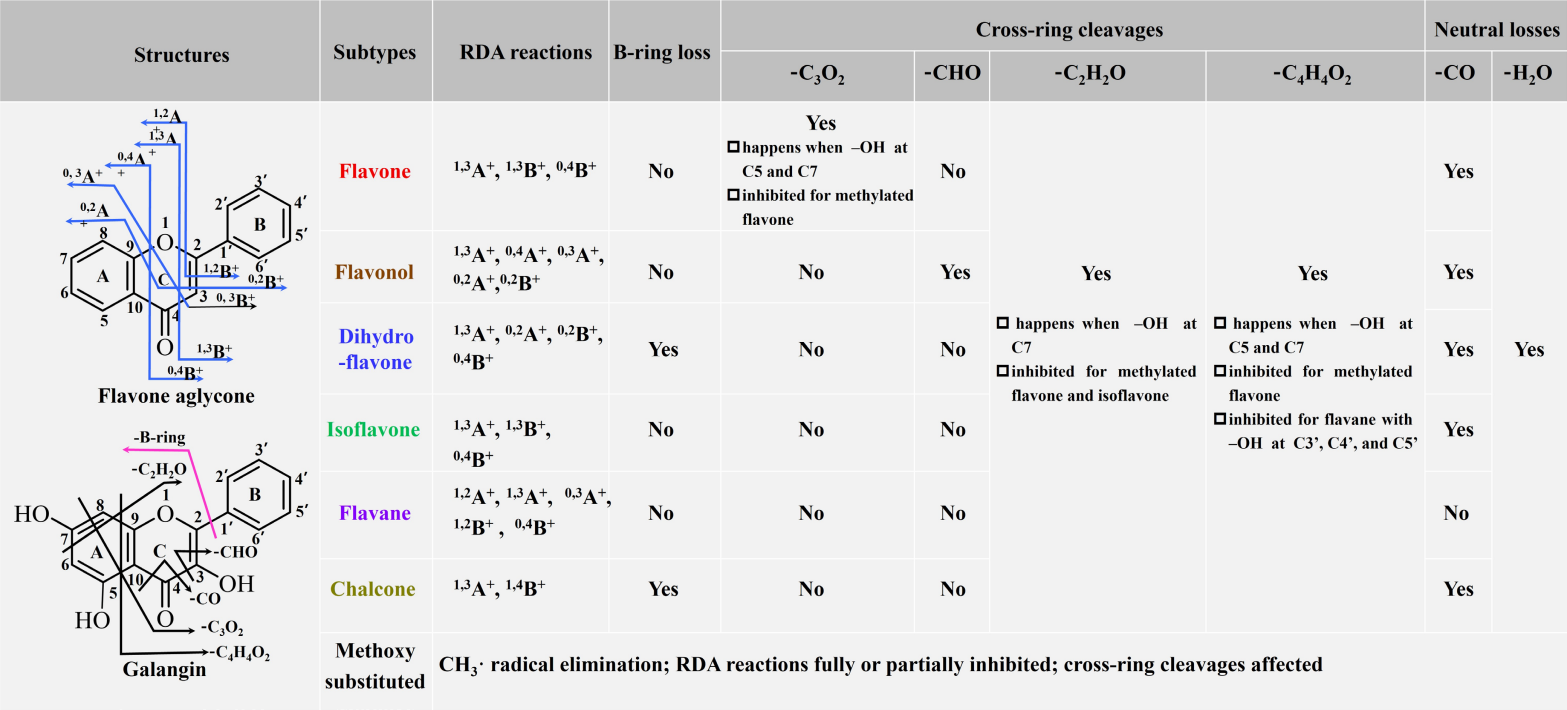
Typical MS^2^ fragmentation rules including RDA reactions, B‐ring loss, cross‐ring cleavages, and neutral losses for different flavonoid subtypes and methoxy substituted flavonoids. Flavone aglycone and galangin are used as examples to show the representative RDA reaction product ions and cross‐ring cleavage pathways to aid the illustration.

With DART‐MS^2^, flavonoids showed some common cleavage patterns in each subclass, and different subtypes exhibited distinctive fragmentation features, which are summarized in Figure 2. Flavone and isoflavone had the same RDA fragments of ^1,3^A^+^, ^1,3^B^+^, and ^0,4^B^+^, but their other features are different. Flavonol and flavane both had 5 RDA reaction products, but they are differentiated by the RDA reaction types. B ring loss was observed for aglycone classes of dihydroflavone and chalcone and can either have a saturated C ring or an open C ring. Only flavone underwent cross‐ring cleavage of C_3_O_2_, and this happened when there are –OH groups on C5 and C7 of the A ring. Moreover, the cleavage of CHO was only found for the flavonol aglycone subclass. Cross‐ring cleavages of C_2_H_2_O and C_4_H_4_O_2_ generally happened for all kinds of flavonoids with specific structural characteristics, and the exceptions are illustrated in Figure 2 as well. Neutral loss of H_2_O and CO was observed for all subtypes, where flavane did not give CO loss since it does not have a CO group in the structure. The features for methoxy‐substituted flavonoids are also included, and they can produce CH_3_· elimination, inhibit some RDA reactions, and affect cross‐ring cleavages.

### 3.2 Flavonoid Isomers

**FIGURE 3:**
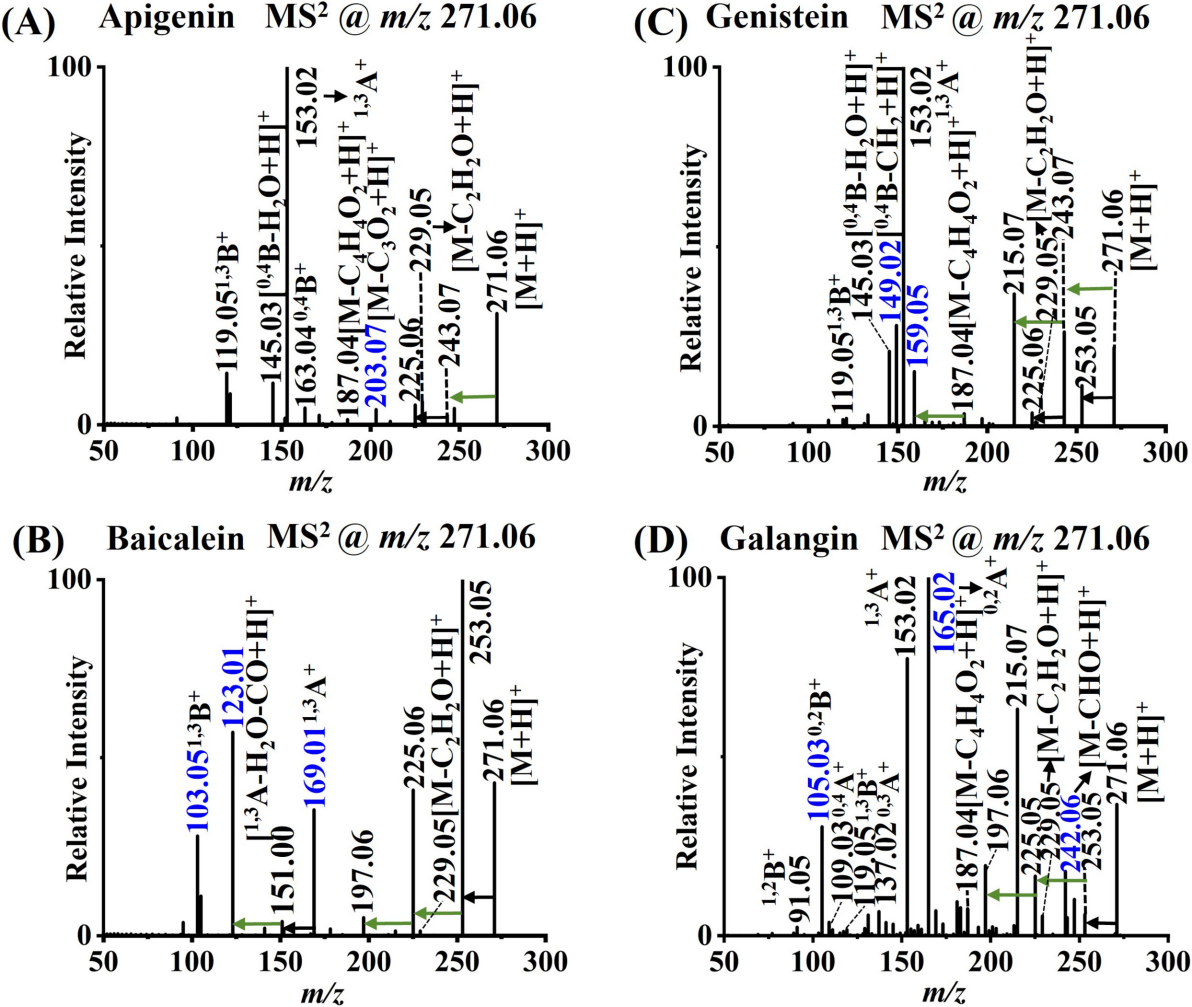
DART‐MS^2^ spectra of flavonoid structural isomers including (A) apigenin, (B) baicalein, (C) genistein, and (D) galangin. Unlabeled black and green arrows indicate mass differences of 18 (H_2_O) and 28 (CO) between peaks, respectively. The *m/z* values of characteristic product ions for each flavonoid are annotated with blue color.

Eight groups of structural isomers from the same or different subtypes were studied. Figure 3 displays the DART‐MS^2^ spectra of four flavonoid structural isomers, namely, apigenin, baicalein, genistein, and galangin. Apigenin and baicalein are both flavone types, and their structures only differ in the –OH group substitution position, while genistein and galangin belong to the isoflavone and flavonol (see Figure 1), respectively. These isomers can undergo very different, similar or even the same RDA reaction pathways. However, due to the stereochemistry differences of flavonoid structural isomers, the same type of RDA reactions can still produce fragments with distinctive *m/z* values. Notably, some RDA reaction ions can continue to lose to produce characteristic product ions such as [^1,3^A–H_2_O–CO + H^+^] ion for baicalein (Figure 3B) and [^0,4^B–CH_2_ + H^+^] fragment for genistein (Figure 3C). In addition, the numbers and patterns of H_2_O and CO losses also varied for different flavonoid isomers, as annotated in Figure 3. As a result, very different MS^2^ spectra were obtained for them using DART ionization. Characteristic fragment ions, uniquely appearing for specific isomer species, were found for each of the isomeric flavonoids, including *m/z* 203.07 for apigenin, *m/z* 103.05, *m/z* 123.01, and *m/z* 169.01 for baicalein, *m/z* 149.02 and *m/z* 159.05 for genistein, and *m/z* 105.03, *m/z* 165.02, and *m/z* 242.06 for galangin. These ions can be used as unique product ions for the identification of flavonoid isomers.

**FIGURE 4:**
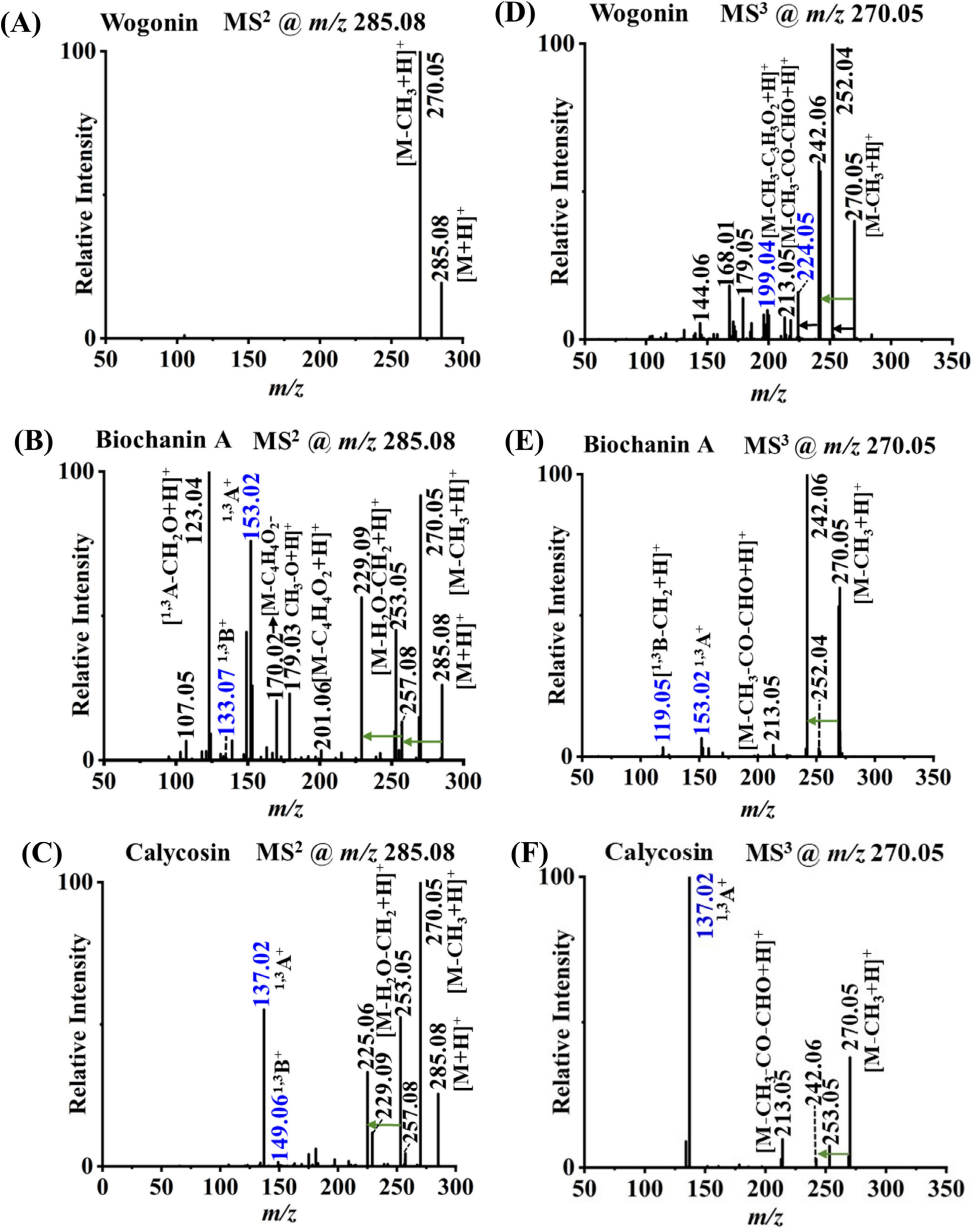
DART‐tandem MS analysis of flavonoid structural isomers including MS^2^ spectra of (A) wogonin, (B) biochanin A, and (C) calycosin, and MS^3^ spectra of (D) wogonin, (E) biochanin A, and (F) calycosin. Unlabeled black and green arrows indicate mass differences of 18 (H_2_O) and 28 (CO) between peaks, respectively. The *m/z* values of characteristic product ions for each flavonoid are annotated with blue color.

Wogonin, biochanin A, and calycosin are structural isomers all having a methoxy substitution. Wogonin belongs to flavone, and biochanin A and calycosin are of the isoflavone type. Biochanin A and calycosin both have two –OH groups but with different substitution positions including C7 and C5 for biochanin A, C7, and C3′ for calycosin (see Figure 1). MS^2^ fragmentation of wogonin resulted in a predominant fragment at *m/z* 270.05 by losing a methyl group (Figure 4A), whereas, in addition to *m/z* 270.05, biochanin A and calycosin produced distinctive MS^2^ ions resulting from RDA reactions, cross‐ring cleavages, and CO losses (Figure 4B,C). For example, characteristic fragments of *m/z* 133.07 (^1,3^B^+^) and 153.02 (^1,3^A^+^) for biochanin A and *m/z* 137.02 (^1,3^A^+^) and 149.06 (^1,3^B^+^) for calycosin were observed. Since no unique MS^2^ product ions were discovered for wogonin, *m/z* 270.05 was further selected for MS^3^ analysis for all three isomers. Wogonin mainly underwent cross‐ring cleavages and neutral losses of H_2_O and CO, resulting in characteristic fragment ions of *m/z* 199.04 (M–CH_3_–C_3_H_3_O_2_ + H^+^) and *m/z* 224.05 (M–CH_3_–CO–H_2_O + H^+^). The diagnostic product ions from MS^3^ analysis for biochanin A and calycosin are still RDA reaction ions and related fragments as indicated in the spectra. Thus, these methoxy‐substituted flavonoid structural isomers can be fully differentiated using featured fragment ions from tandem MS using DART ionization, without any sample derivatization or liquid phase separation.

DART‐tandem MS spectra of additional flavonoid structural isomers are shown in Figures S1–S7 with discussions in the Supporting Information. Various flavonoid structural isomers can generate distinguishable fragmentation spectra with unique characteristic product ions. To test the capability of identifying specific flavonoid compounds from complex matrixes, DART‐MS^2^ analysis was performed for a mixture of 14 flavonoid compounds including 5 groups of structural isomers in Figure S8. Five different mass signals were obtained from the MS scan, and they correspond to the molecular ions of five groups of flavonoid structural isomers. To further differentiate these isomers, DART‐MS^2^ was further conducted on different precursor ions. According to their fragmentation spectra, characteristic product ions of different isomers were generated for each flavonoid isomer, and they can be directly identified from the mixed sample. The spectra obtained from the mixture are consistent with their individual compounds, and the ionization behaviors do not change when they are put together.

### 3.3 Workflow for Characterization of Flavonoid Structures

**FIGURE 5:**
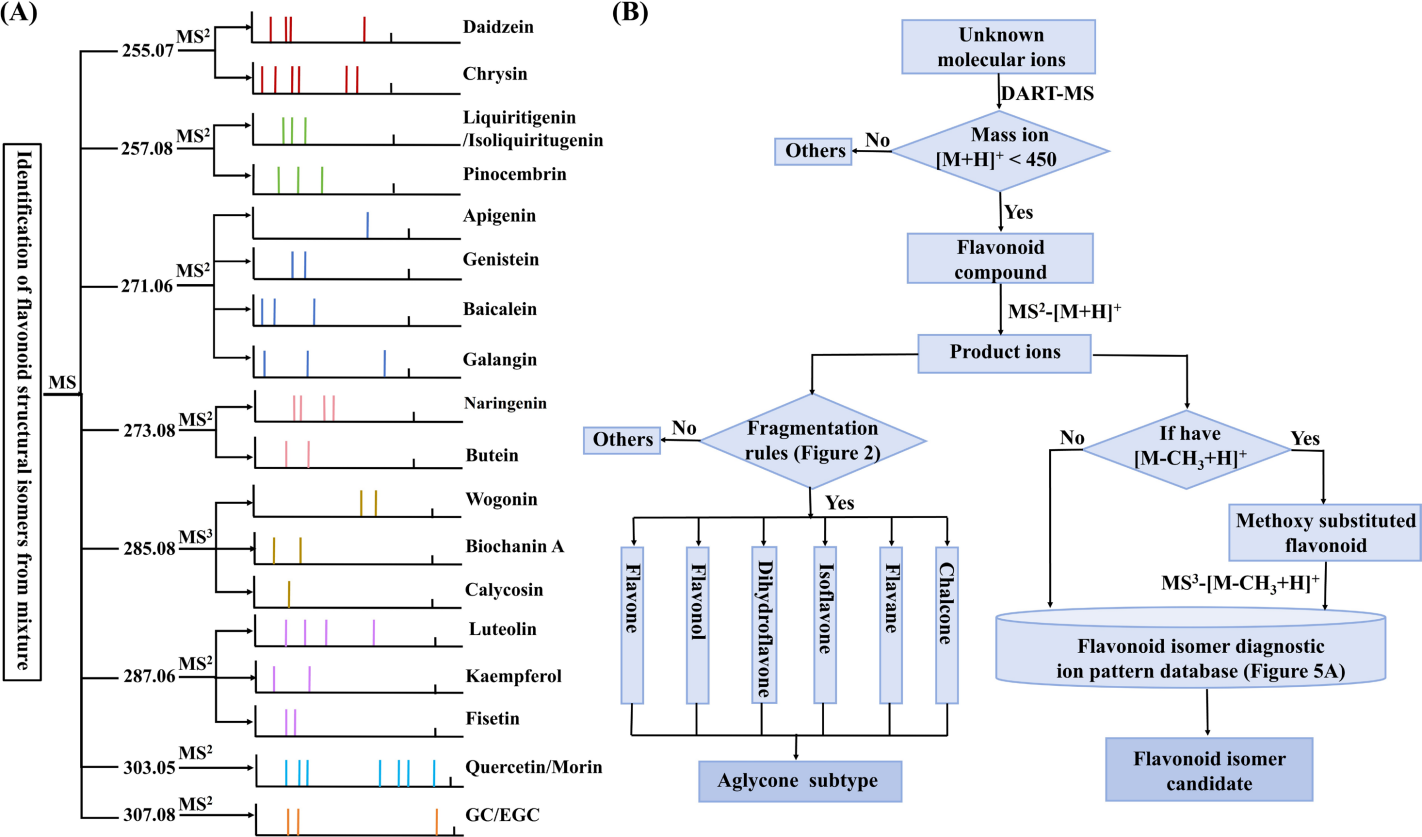
(A) Schematic diagram of identification of flavonoid structural isomers from mixture using diagnostic product ion patterns from DART‐tandem MS analysis. (B) Workflow for the determination of flavonoid aglycone subtype and structural isomer following DART‐tandem MS.

A schematic diagram for the identification of different flavonoid structural isomers using the unique product ions from mixtures is shown in Figure 5A. The chart included 8 groups of isomers of 21 flavonoid compounds. In the initial MS scan, the flavonoids with the same molecular formula all produce the same signals, resulting in eight molecular ions for the flavonoids at *m/z* 255.07, *m/z* 257.08, *m/z* 271.06, *m/z* 273.08, *m/z* 285.08, *m/z* 287.06, *m/z* 303.05, and *m/z* 307.08, respectively. To characterize a specific isomer, these molecular ions were chosen for tandem MS analysis. The identity of the product ions of different flavonoid isomers is represented by sticks at different positions and with different colors in the normalized spectra, resulting in a very distinctive recognition map of individual isomer(s). The detailed fragment ions are shown in Figure S9. For flavonoids without methoxy substitution, DART‐MS^2^ was used to generate the characteristic ions of the corresponding isomers. However, for methylated flavonoids, overlapping of signals could occur due to CH_3_· elimination from DART‐MS^2^ analysis; therefore, DART‐MS^3^ analysis of [M–CH_3_ + H^+^] is needed. Relying on the characteristic product ion pattern of each isomer, different flavonoids with subtle structural variations can be directly and rapidly identified without chromatographic separations.

From the structural analysis of flavonoid aglycone subtypes and isomers in this work, a determination tree is developed in Figure 5B to recognize flavonoid aglycone subtypes and isomeric features. Based on the compounds studied in this work and reported in the literature, the molecular weights of flavonoid compounds are generally below 410 [18, 27, 43]. For an unknown compound, *m/z* of 450 from DART‐MS analysis can be used as a cutoff line for distinguishing flavonoid candidates from other compounds. DART‐MS^2^ analysis was then performed by CID experiments to molecular ions to obtain the fragments. The product ions can be arranged into different categories including RDA reactions, cross‐ring cleavages, B‐ring loss and neutral losses. These patterns will be examined by the flavonoid subtype fragmentation rules established in Figure 2. According to the compliant situations, a specific aglycone class can be determined or assigned to others. On the other hand, by screening a mass difference of 15.02 between product ions, the methoxy‐substituted flavonoids can be recognized. A further MS^3^ analysis will be performed to [M–CH_3_ + H^+^] ions to obtain adequate fragment ions. If no [M–CH_3_ + H^+^] is discovered, MS^3^ analysis will not be conducted. Ultimately, all the tandem MS signals will be imported into the diagnostic ion database of flavonoid structural isomers, and based on the unique characteristic fragment pattern in Figure 5A, structural profiling of a specific flavonoid isomer can be achieved. This workflow is a powerful new tool using ambient ionization and tandem MS analysis for the detailed structural characterization of flavonoid compounds.

According to the determination workflow including featured fragmentation rules and diagnostic product ions, we created a software called FlavoFinder with two functional modules of flavonoid subtype and flavonoid isomer (http://www.flavofinder.com). It incorporates an automated data analysis program to predict the flavonoid structure using DART‐MS and tandem MS ions, and provides a pathway for structural elucidation using ambient ionization MS for other compounds as well. The in‐house database, which contains diagnostic product ions and decision path, will be continuously updated and expanded to enhance its functionality and coverage for compound annotation.

### 3.4 Quantitative Analysis of Flavonoid Isomers in Real Samples

**FIGURE 6:**
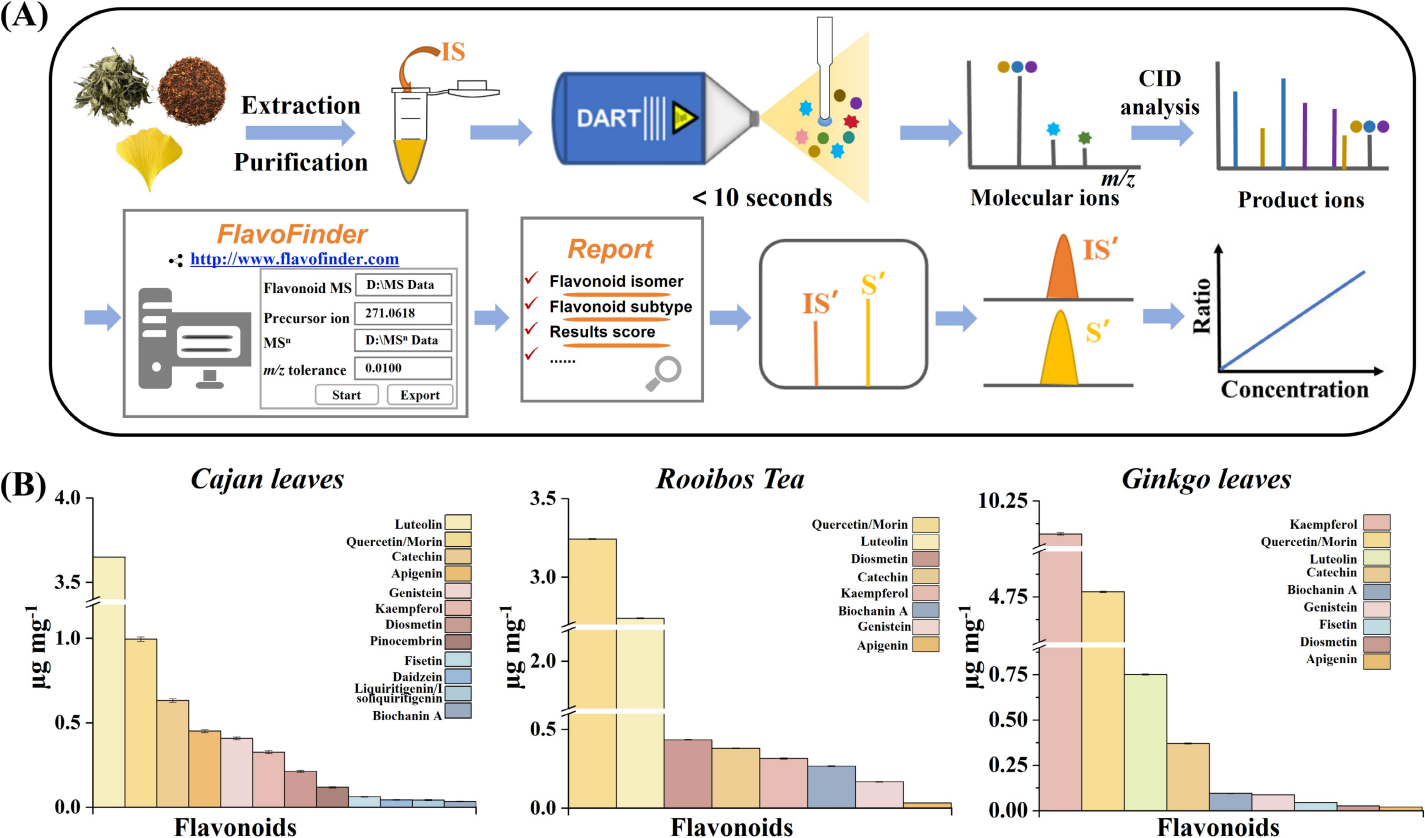
Qualitative and quantitative analysis of flavonoid compounds in real samples using DART‐tandem MS analysis including (A) identification workflow and (B) measured contents of different flavonoids in cajan leaves, rooibos tea, and ginkgo leaves, respectively.

Following the procedure in Figure 6A, three real‐life samples including cajan leaves, rooibos tea and ginkgo leaves were extracted and purified, and the processed samples were added with internal standard (IS) and subjected to DART tandem MS analysis. Qualitative identification of different flavonoids can be achieved based on the schematic diagram and workflow established in this work. Figure S10 shows full mass scan and MS^2^ and MS^3^ spectra of cajan leaves using DART ion source. A number of molecular ions appeared in the *m/z* range from 150 to 650. To achieve isomeric compound identification, different molecular ions were selected for CID experiments. According to the unique product ions, individual flavonoid compounds can be identified as annotated. DART‐tandem MS analysis of rooibos tea and ginkgo leaves was also performed following the same procedures, and the results are shown in Figures S11 and S12, respectively. At the same time, the full MS scan and MS^2^ and MS^3^ peak lists were imported to FlavoFinder program, and the FlavoReports were obtained instantaneously. The manual analysis results are consistent with the software‐generated reports.

Quantification was achieved by comparing the peak area ratios of identified flavonoids' diagnostic product ions and fragment of IS with established calibration curves. Calibration curves of 16 flavonoid compounds from different subtypes were established and are shown in Figure S13. To minimize the influence of the ambient environment and sampling conditions for DART ionization, an IS of acetyl rimantadine was added to each standard solution. A series of flavonoid concentrations were used as the X axis, and the extracted peak area ratios of the characteristic product ions of the flavonoid and the fragment of the IS from DART‐MS^2^ analysis were used as the Y axis. The linear correlation, reproducibility, limit of detection (LOD), and limit of quantitation (LOQ) are summarized in Table S2. The linear correlation coefficients ranged from 0.9902 to 0.9993, and the relative standard deviations (RSDs) ranged from 5.19% to 16.30%. Owing to the ambient ionization nature of the DART ion source, its analytical results typically exhibit higher RSD values [44, 45] than those obtained by LC–MS [46, 47]. The use of an isotope‐labelled IS would be critical to further improve the reproducibility and precision of DART analyses [48]. The LODs and LOQs of 16 flavonoids were obtained in the range from 0.2 to 524.6 ng mL^−1^ and from 0.7 to 1748.6 ng mL^−1^, respectively.

As a result, the identified flavonoid species with their quantitative information are shown in Figure 6B. In this case, 14 flavonoids for cajan leaves, 9 for rooibos tea, and 10 for ginkgo leaves were discovered. Some flavonoid species are detected in all three real samples, but others are different. Among these compounds, specific isomeric forms were successfully identified, for example, apigenin, genistein, luteolin, kaempferol, and fisetin. Flavonoids of daidzein, liquiritigenin, and/or isoliquiritigenin and pinocembrin were only found in cajan leaves, and not in rooibos tea and ginkgo leaves. Except for fisetin, the flavonoid species detected in ginkgo leaves are the same as those in rooibos tea. The highest abundance of flavonoids in both cajan leaves and rooibos tea are luteolin and quercetin/morin. Ginkgo leaves have the highest amount of kaempferol, which is about 20 times more than kaempferol found in cajan leaves and rooibos tea. With the method developed in this work, the qualitative and quantitative variations of flavonoid species in different plant samples were successfully measured within a few minutes.

The DART‐MS method developed in this work offers drastically shorter analysis times than conventional LC–MS [49, 50, 51]. Moreover, while LC–MS remains a powerful tool for comprehensive profiling, our results demonstrate the distinct advantage of DART‐MS in the differentiation of isomeric flavonoids. This was achieved by using diagnostic product ions for both qualitative and quantitative analysis, which provides high selectivity against matrix backgrounds and enables rapid, chromatography‐free discrimination of isomers in standard mixtures and real samples. It should be noted, however, that applying this method to highly complex samples may still be challenged by significant matrix interference. Thus, appropriate sample purification or enrichment steps prior to analysis are necessary to reduce spectral complexity and ensure reliable and accurate results.

## 4 Conclusions

In this work, a method using DART ionization tandem MS for the structural determination and profiling of flavonoid compounds was established, where flavonoid aglycone subtypes and structural isomers can both be identified. Each class of flavonoids exhibited specific fragmentation features from RDA reactions, cross‐ring cleavages, and neutral losses, and these features can be used to recognize aglycone subtypes. Methoxy substitution gave significant methyl group loss, and can inhibit some RDA reactions and cross‐ring cleavages. This is important for the structural elucidation of different flavonoid skeletons from complex mixtures. A number of flavonoid structural isomers of different and/or same subclasses showed distinctive fragmentation patterns, and unique product ions of different isomers were obtained and tabulated. The isomer identification was successfully validated in the analysis of flavonoid mixtures using these unique characteristic fragment ions. This workflow is capable of elucidating flavonoid aglycone and isomer information following MS^2^ (for non‐methylated) and MS^3^ (for methylated flavonoids) analysis using DART ionization. A software tool was developed for ambient ionization and tandem MS structural elucidation, to achieve automatic data analysis for flavonoid structure annotation. Flavonoid species in complex matrices of real samples were qualitatively and quantitatively determined using the developed method, and the identification was validated with a computer‐generated report.

The analysis process can be completed in a much shorter time than any other currently used methodology, and with much better results. Without the uncertainty caused by the front‐end liquid or gas phase separations, tandem MS product ions play crucial roles in the structural characterization process, which is more accurate and reliable compared to other factors, for example, UV absorption maxima, or retention times from varied GC or LC conditions. This method has significant advantages in terms of simplicity, speed and robustness over traditional tedious and time‐consuming analytical techniques for the successful characterization of multiple flavonoid isomers from real‐life samples.

## Author Contributions

**Yanqiu Wang:** data curation, formal analysis, methodology, software, validation, writing – original draft. **Liping Xu:** data curation, formal analysis, investigation, methodology, validation, visualization. **Tiange Gu:** methodology, software. **Hongli Li:** conceptualization, funding acquisition, project administration, supervision, writing – review and editing. **David Da Yong Chen:** funding acquisition, project administration, resources, writing – review and editing.

## Conflicts of Interest

The authors declare no conflicts of interest.

## Supporting information


**Figure S1:** DART‐MS^2^ spectra of flavonoid structural isomers of (A) daidzein and (B) chrysin. Unlabeled black and green arrow indicate mass differences of 18 (H_2_O) and 28 (CO) between peaks, respectively. The *m/z* values of characteristic product ions for each flavonoid are annotated with blue color.
**Figure S2:** DART‐MS^2^ spectra of flavonoid structural isomers of (A) pinocembrin, (A) liquiritigenin, and (C) isoliquiritigenin. Unlabeled black and green arrows indicate mass differences of 18 (H_2_O) and 28 (CO) between peaks, respectively. The *m/z* values of characteristic product ions for each flavonoid are annotated with blue color.
**Figure S3:** DART‐MS^2^ spectra of flavonoid structural isomers of (A) naringenin and (B) butein. Unlabeled black and green arrow indicate mass differences of 18 (H_2_O) and 28 (CO) between peaks, respectively. The *m/z* values of characteristic product ions for each flavonoid are annotated with blue color.
**Figure S4:** DART‐MS^2^ spectra of flavonoid structural isomers including (A) luteolin, (B) kaempferol, and (C) fisetin. Unlabeled black and green arrows indicate mass differences of 18 (H_2_O) and 28 (CO) between peaks, respectively. The *m/z* values of characteristic product ions for each flavonoid are annotated with blue color. MS/MS and MS^2^ indicate the same method with different writing presentations.
**Figure S5:** DART‐MS^2^ spectra of flavonoid structural isomers of (A) quercetin and (B) morin. Unlabeled black and green arrows indicate mass differences of 18 (H_2_O) and 28 (CO) between peaks, respectively.
**Figure S6:** DART‐MS^2^ spectra of flavonoid structural isomers of (A) gallocatechin (GC), and (B) epigallocatechin (EGC). Unlabeled black arrows indicate mass differences of 18 (H_2_O).
**Figure S7:** DART‐MS^2^ spectra for other flavonoid compounds including (A) 4‐methoxychalcone, (B) phloretin, (C) catechin, (D) diosmetin, (E) hesperetin, and (F) myricetin. Unlabeled black and green arrows indicate mass differences of 18 (H_2_O) and 28 (CO) between peaks, respectively.
**Figure S8:** DART‐MS^2^ analysis of mixture of five groups of flavonoid isomers including daidzein and chrysin (MW 254.06), liquiritigenin and pinocembrin (MW 256.07), apigenin, genistein, baicalein, and galangin (MW 270.05), wogonin, biochanin A, and calycosin (MW 284.07), and luteolin, kaempferol, and fisetin (MW 286.05). (A) DART‐MS analysis of flavonoid isomer mixtures, DART‐MS^2^ analysis for molecular ion of (B) *m/z* 255.07, (C) *m/z* 257.08, (D) *m/z* 271.06, (E) *m/z* 285.08, and (F) *m/z* 287.06, respectively. MW indicates molecular weight.
**Figure S9:** Schematic diagram of identification of flavonoid structural isomer from mixture using specific product ions obtained for each compound from DART‐MS^n^ analysis.
**Figure S10:** Full mass scan and DART‐DART‐MS^n^ analysis of cajan leaf extract. The identifications of different flavonoid compound are indicated on the spectra.
**Figure S11:** Full mass scan and DART‐DART‐MS^n^ analysis of rooibos tea extract. The identifications of different flavonoid compound are indicated on the spectra.
**Figure S12:** Full mass scan and DART‐DART‐MS^n^ analysis of ginkgo leaf extract. The identifications of different flavonoid compound are indicated on the spectra.
**Figure S13:** Calibration curves of 16 flavonoid compounds using DART‐MS^2^ analysis. A series concentrations of standard solutions were used as x‐axis, and the extracted peak area ratios of diagnostic product ion from flavonoid and fragment of IS (*m/z* 163) were used as y axis.
**Table S1:** List of flavonoid standards in this work with structural characteristics.
**Table S2:** The linear correlation coefficients, LODs, LOQs and RSDs for the 16 flavonoids.

## Data Availability

Data will be made available on request.
