# Supplementary material for: Direct Identification and Quantification of Flavonoids and Their Structural Isomers Using Ambient Ionization Tandem Mass Spectrometry
Source: Rapid Commun Mass Spectrom. 2025 Nov 8;40(3):e10169. doi: 10.1002/rcm.10169 (PMC12595406; doi:10.1002/rcm.10169)
Supplement: Supplementary file 1 — Figure S1: DART‐MS2 spectra of flavonoid structural isomers of (A) daidzein and (B) chrysin. Unlabeled black and green arrow indicate mass differences of 18 (H2O) and 28 (CO) between peaks, respectively. The m/z values of characteristic product ions for each flavonoid are annotated with blue color. Figure S2: DART‐MS2 spectra of flavonoid structural isomers of (A) pinocembrin, (A) liquiritigenin, and (C) isoliquiritigenin. Unlabeled black and green arrows indicate mass differences of 18 (H2O) and 28 (CO) between peaks, respectively. The m/z values of characteristic product ions for each flavonoid are annotated with blue color. Figure S3: DART‐MS2 spectra of flavonoid structural isomers of (A) naringenin and (B) butein. Unlabeled black and green arrow indicate mass differences of 18 (H2O) and 28 (CO) between peaks, respectively. The m/z values of characteristic product ions for each flavonoid are annotated with blue color. Figure S4: DART‐MS2 spectra of flavonoid structural isomers including (A) luteolin, (B) kaempferol, and (C) fisetin. Unlabeled black and green arrows indicate mass differences of 18 (H2O) and 28 (CO) between peaks, respectively. The m/z values of characteristic product ions for each flavonoid are annotated with blue color. MS/MS and MS2 indicate the same method with different writing presentations. Figure S5: DART‐MS2 spectra of flavonoid structural isomers of (A) quercetin and (B) morin. Unlabeled black and green arrows indicate mass differences of 18 (H2O) and 28 (CO) between peaks, respectively. Figure S6: DART‐MS2 spectra of flavonoid structural isomers of (A) gallocatechin (GC), and (B) epigallocatechin (EGC). Unlabeled black arrows indicate mass differences of 18 (H2O). Figure S7: DART‐MS2 spectra for other flavonoid compounds including (A) 4‐methoxychalcone, (B) phloretin, (C) catechin, (D) diosmetin, (E) hesperetin, and (F) myricetin. Unlabeled black and green arrows indicate mass differences of 18 (H2O) and 28 (CO) between peaks, respectiv [file RCM-40-e10169-s001.docx]

**Supplementary Information for:**

**Direct Identification and Quantification of Flavonoids and Their Structural Isomers using Ambient Ionization Tandem Mass Spectrometry**

Yanqiu Wang ^1, †^ | Liping Xu ^1, †^ | Tiange Gu ^1^ | Hongli Li ^1,*^ | David Da Yong Chen ^1,2,3,*^

^1^ Jiangsu Collaborative Innovation Center of Biomedical Functional Materials, Jiangsu Key Laboratory of Biomedical Materials, School of Chemistry and Materials Science, Nanjing Normal University, Nanjing 210023, China

^2^ Department of Chemistry, University of British Columbia, Vancouver, BC, Canada V6T 1Z1

^3^ State Key Laboratory of Analytical Chemistry for Life Science, School of Chemistry and Chemical Engineering, Nanjing University, Nanjing 210093, China

^†^These authors contributed equally to this work

^*^Corresponding author: E-mail address: [lihongli@njnu.edu.cn](mailto:lihongli@njnu.edu.cn) (H. Li), chen@chem.ubc.ca (D. D. Y. Chen). Tel.: +86 13605172445

**Table of Contents**

**Figure S1.** DART-MS^2^ spectra of daidzein and chrysin…….….…...…………Page S3

**Figure S2.** DART-MS^2^ spectra of pinocembrin, liquiritigenin and isoliquiritigenin….…….…….…….…….…….…….…….…….…….…….…Page S4

**Figure S3.** DART-MS^2^ spectra of naringenin and butein.….….….......………Page S5

**Figure S4.** DART-MS^2^ spectra of luteolin, kaempferol and fisetin....................Page S6

**Figure S5.** DART-MS^2^ spectra of quercetin and morin………………………..Page S7

**Figure S6.** DART-MS^2^ spectra of GC and EGC...……………………………..Page S8

**Figure S7.** DART-MS^2^ spectra of non-isomeric flavonoids…………………...Page S9

**Figure S8.** DART-MS^2^ analysis of mixture…………………………………..Page S10

**Figure S9.** Flavonoid isomers with diagnostic fragments ……………….…..Page S11

**Figure S10.** DART-MS^n^ analysis of cajan leaf extract.………………............Page S12

**Figure S11.** DART- MS^n^ analysis of rooibos tea extract……………..............Page S12

**Figure S12.** DART- MS^n^ analysis of ginkgo leaf extract.…………….............Page S13

**Figure S13.** Calibration curves of 16 flavonoid compounds ………………...Page S14

**Table S1.** List of flavonoid standards with structural characteristics………...Page S15

**Table S2.** Linear correlation coefficients, LODs, LOQs and RSDs………….Page S16

**
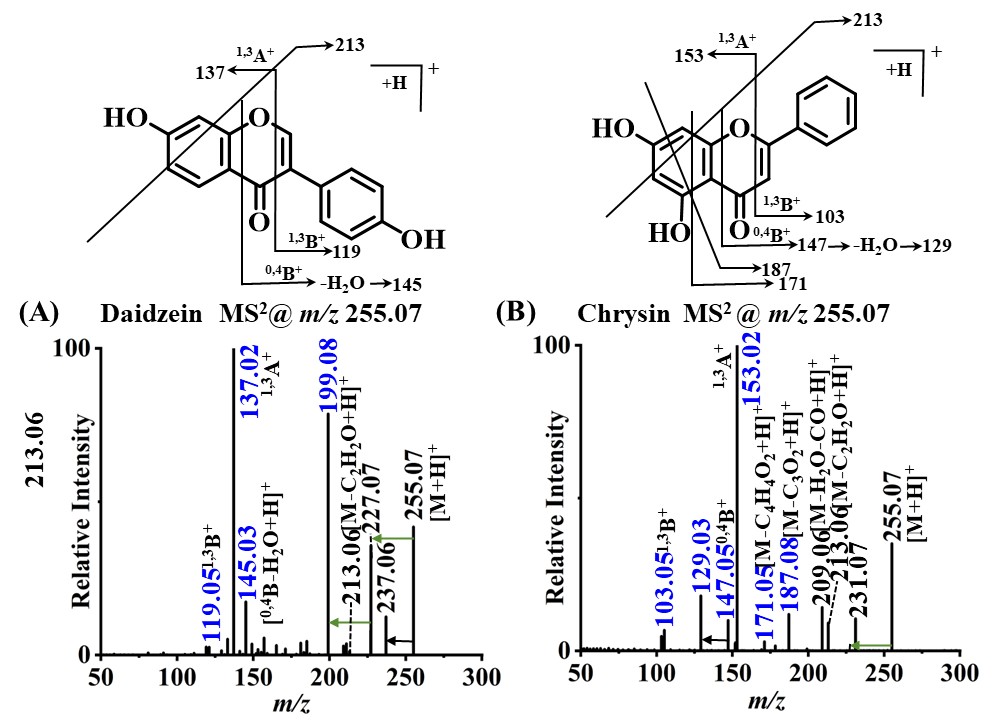
**

**Figure S1.** DART-MS^2^ spectra of flavonoid structural isomers of (A) daidzein, and (B) chrysin. Unlabeled black and green arrow indicate mass differences of 18 (H_2_O) and 28 (CO) between peaks, respectively. The *m/z* values of characteristic product ions for each flavonoid are annotated with blue color.

**Discussion for Figure S1:**  Flavonoid isomers of daidzein and chrysin belong to isoflavone and flavone, respectively. They both have 2 hydroxyl groups, but with different substitution sites. Daidzein and chrysin both exhibited RDA cleavages of ^1,3^A^+^, ^1,3^B^+^, ^0,4^B^+^, but these fragments are produced with characteristic *m/z* values due to their structure variations. In addition, chrysin has a cross-ring cleavages of C_3_O_2_ and C_4_H_4_O_2_, which were not observed for daidzein.


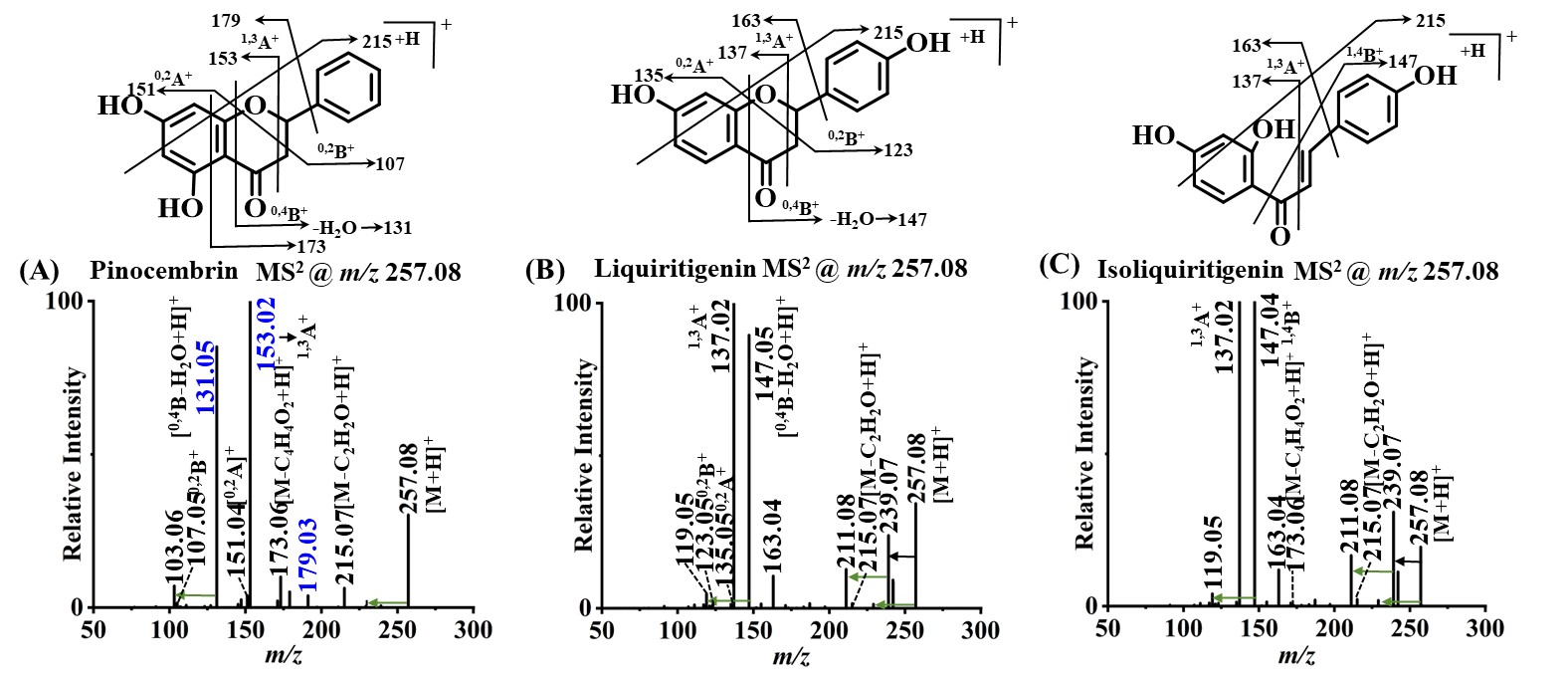


**Figure S2.** DART-MS^2^ spectra of flavonoid structural isomers of (A) pinocembrin, (A) liquiritigenin, and (C) isoliquiritigenin. Unlabeled black and green arrows indicate mass differences of 18 (H_2_O) and 28 (CO) between peaks, respectively. The *m/z* values of characteristic product ions for each flavonoid are annotated with blue color.

**Discussion for Figure S2:** Pinocembrin and liquiritigenin are both of dihydroflavone type, and their structure difference only lies in the position of a single –OH group, while isoliquiritigenin belongs to chalcone. The fragmentation pathways for pinocembrin and liquiritigenin using DART ion source are very similar. However, owing to their stereochemical difference, they generated significantly different product ions (Figure S2A, B). Although the RDA reactions of liquiritigenin and isoliquiritigenin are different, the fragment signals produced are essentially the same and cannot be differentiated (Fig. S2C).

**
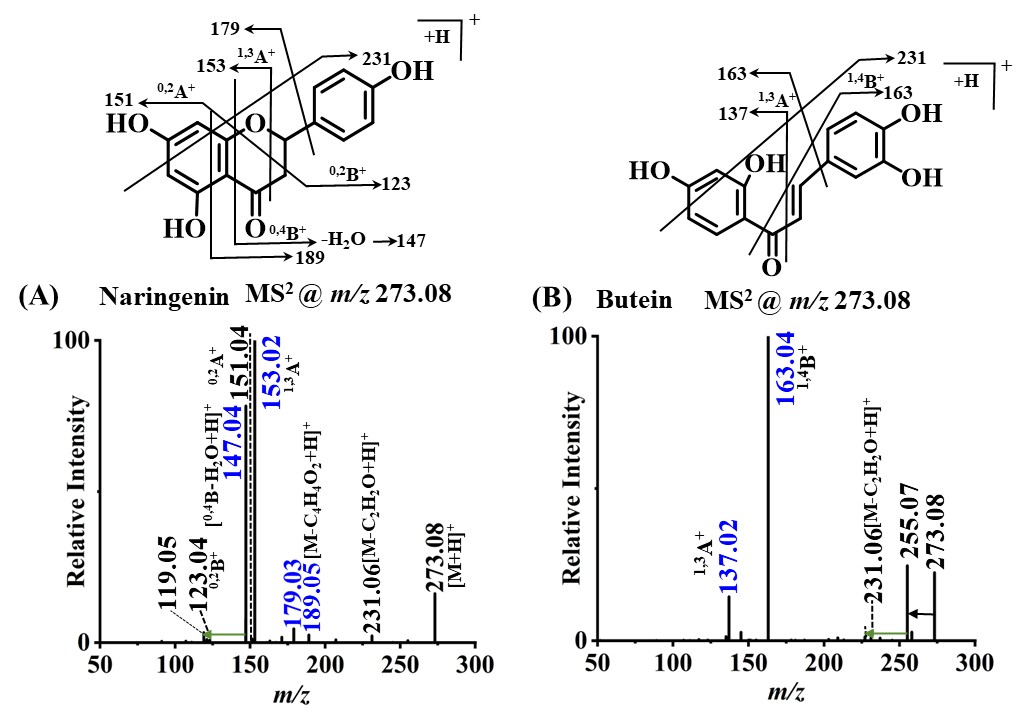
**

**Figure S3.** DART-MS^2^ spectra of flavonoid structural isomers of (A) naringenin, and (B) butein. Unlabeled black and green arrow indicate mass differences of 18 (H_2_O) and 28 (CO) between peaks, respectively. The *m/z* values of characteristic product ions for each flavonoid are annotated with blue color.

**Discussion for Figure S3:** Isomers of naringenin and butein belong to dihydroflavone and chacone, respectively. Naringenin produced abundant RDA fragments of ^0,2^A^+^, ^1,3^A^+^, ^0,2^B^+^, ^0,4^B^+^, and underwent cross-ring cleavages of C_4_H_4_O_2_ and C_2_H_2_O, while butein only showed apparent RDA reactions of ^1,3^A^+^ and ^1,4^B^+^, and cross-ring cleavage of C_2_H_2_O, leading to the successful identification with DART-MS^2^.


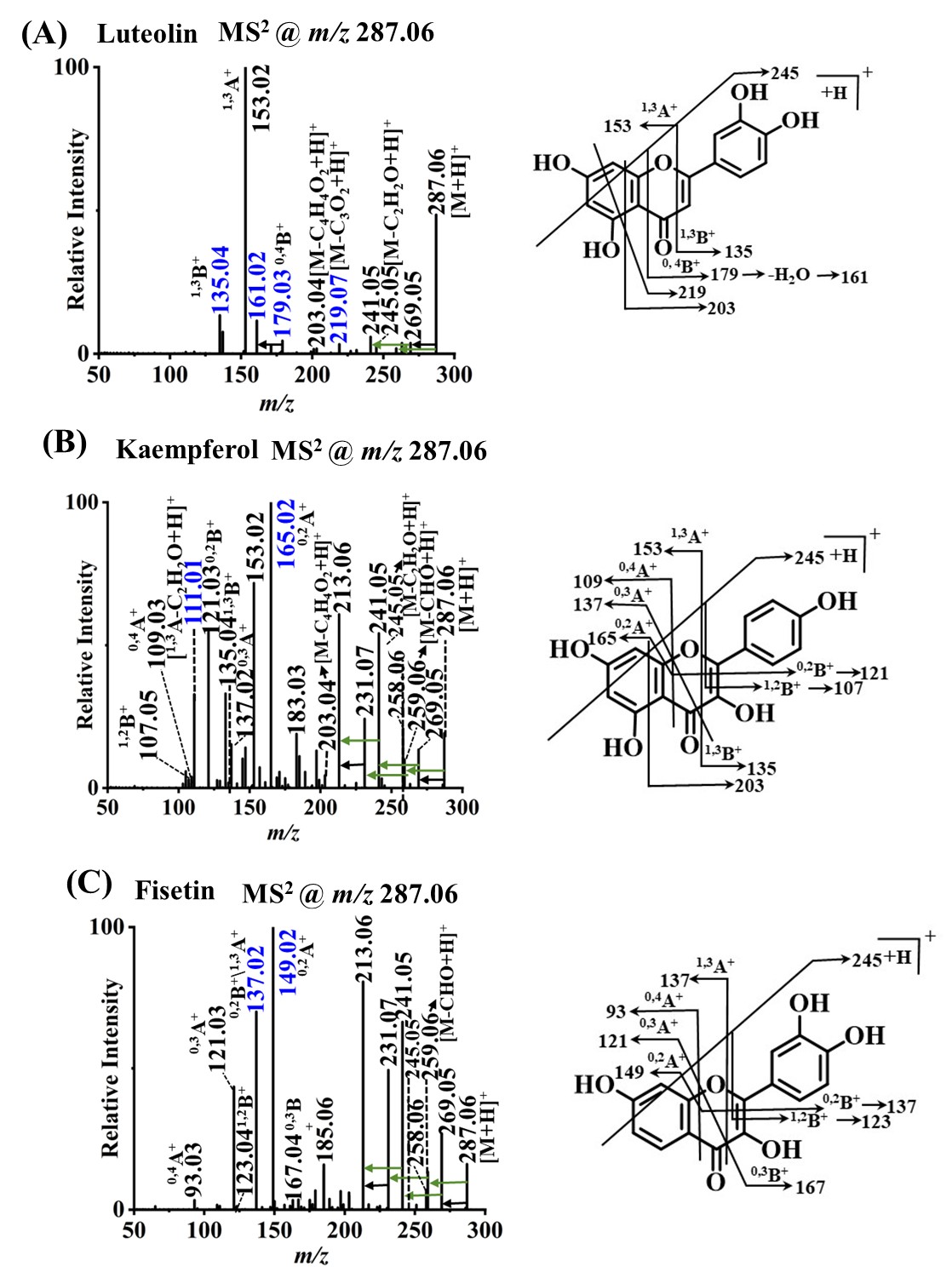


**Figure S4.** DART-MS^2^ spectra of flavonoid structural isomers including (A) luteolin, (B) kaempferol, and (C) fisetin. Unlabeled black and green arrows indicate mass differences of 18 (H_2_O) and 28 (CO) between peaks, respectively. The *m/z* values of characteristic product ions for each flavonoid are annotated with blue color. MS/MS and MS^2^ indicate the same method with different writing presentations.

**Discussion for Figure S4:** Luteolin of flavone type, and kaempferol and fisetin from flavonol subclass are flavonoid structural isomers having molecular ion at *m/z* 287.06. They all have four –OH groups but with different attachment sites. Very different fragmentation spectra were observed owing to different RDA reaction products, cross-ring cleavages, and neutral losses, leading to distinctive ions for each isomer. MS/MS and MS^2^ indicate the same method with different writing presentations.


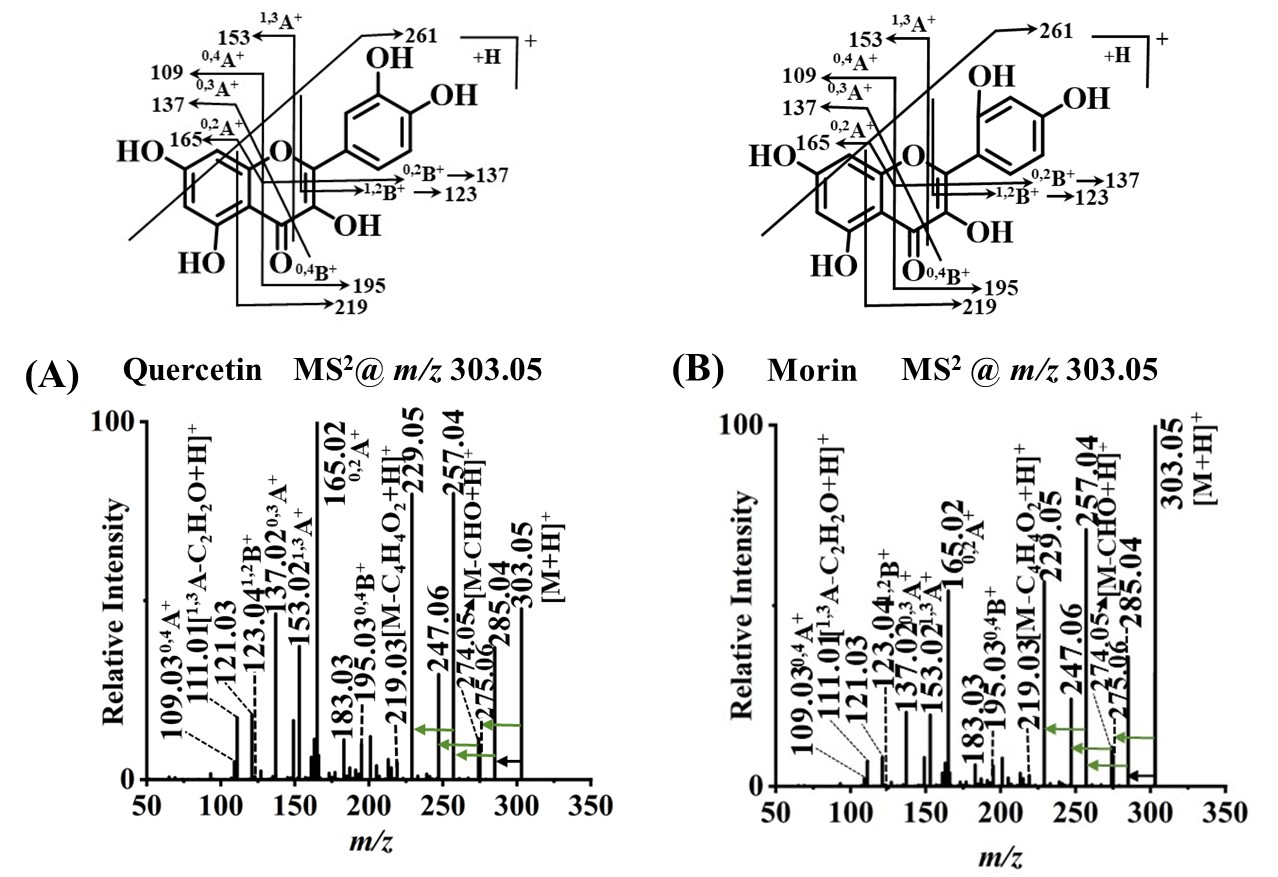


**Figure S5.** DART-MS^2^ spectra of flavonoid structural isomers of (A) quercetin, and (B) morin. Unlabeled black and green arrows indicate mass differences of 18 (H_2_O) and 28 (CO) between peaks, respectively.


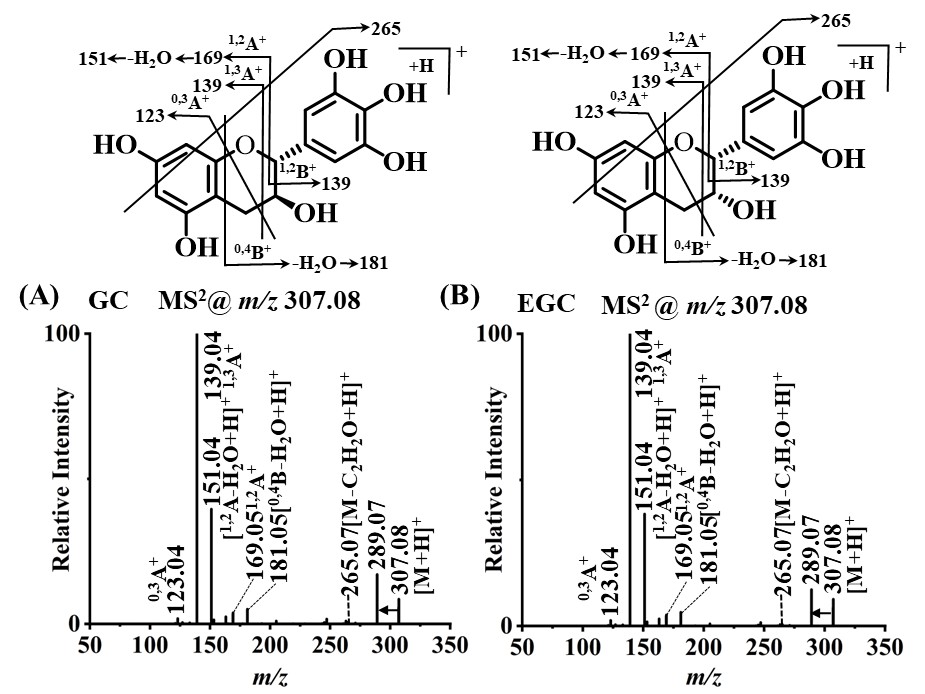


**Figure S6.** DART-MS^2^ spectra of flavonoid structural isomers of (A) gallocatechin (GC), and (B) epigallocatechin (EGC). Unlabeled black arrows indicate mass differences of 18 (H_2_O).


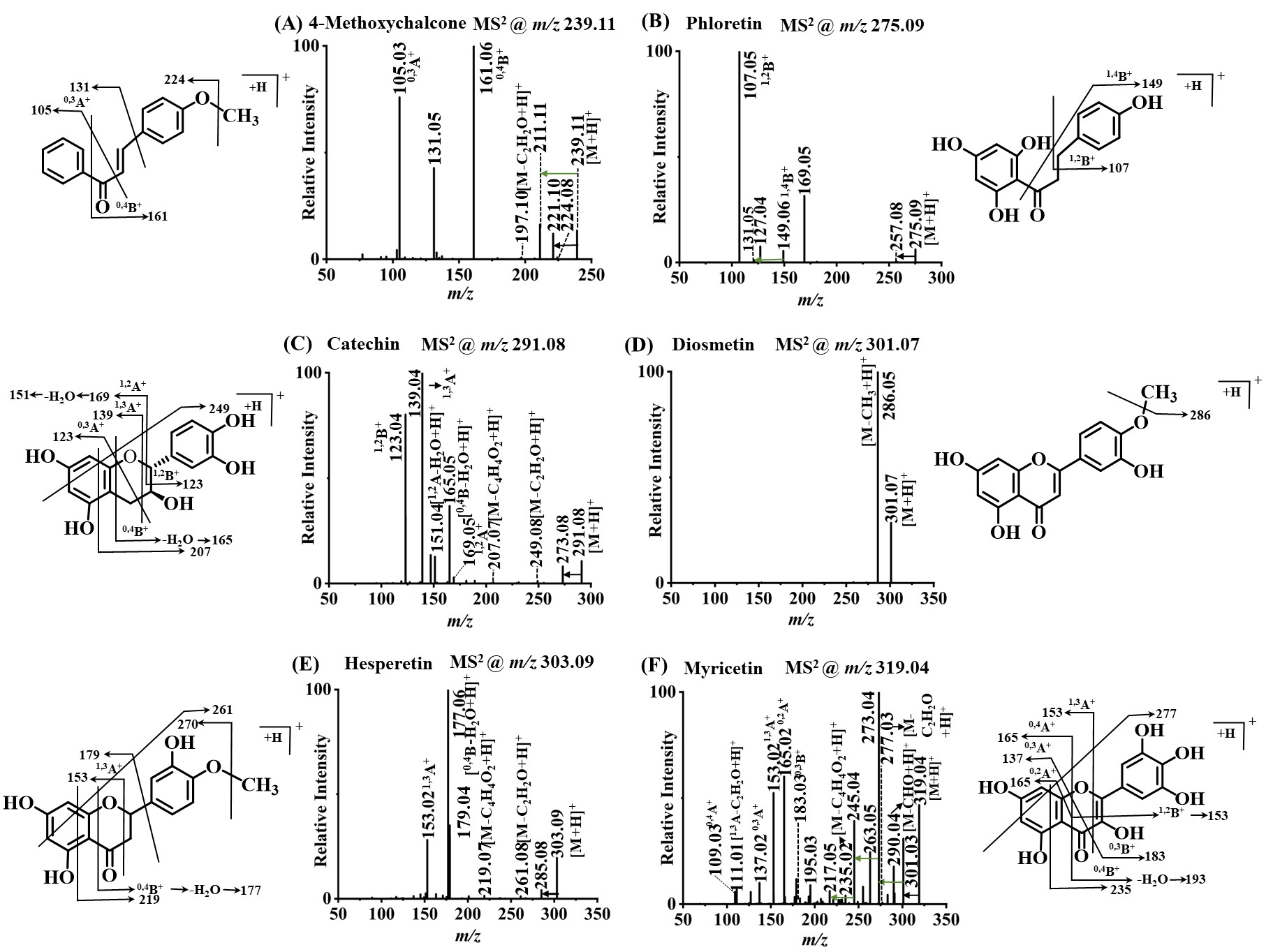


**Figure S7.** DART-MS^2^ spectra for other flavonoid compounds including (A) 4-methoxychalcone, (B) phloretin, (C) catechin, (D) diosmetin, (E) hesperetin and (F) myricetin. Unlabeled black and green arrows indicate mass differences of 18 (H_2_O) and 28 (CO) between peaks, respectively.

**Discussion for Figures S5-S7:** In addition, flavonol isomers of quercetin and morin, and flavane isomers of GC and EGC have very subtle stereochemical structure differences. Their DART-MS^2^ spectra are shown in Fig. S5 and Fig. S6, respectively. The resulted fragmentation patterns are very similar and these two pairs of isomers cannot be distinguished. The DART-MS^2^ spectra for other non-isomerized flavonoid compounds are shown in Fig. S7 for reference.


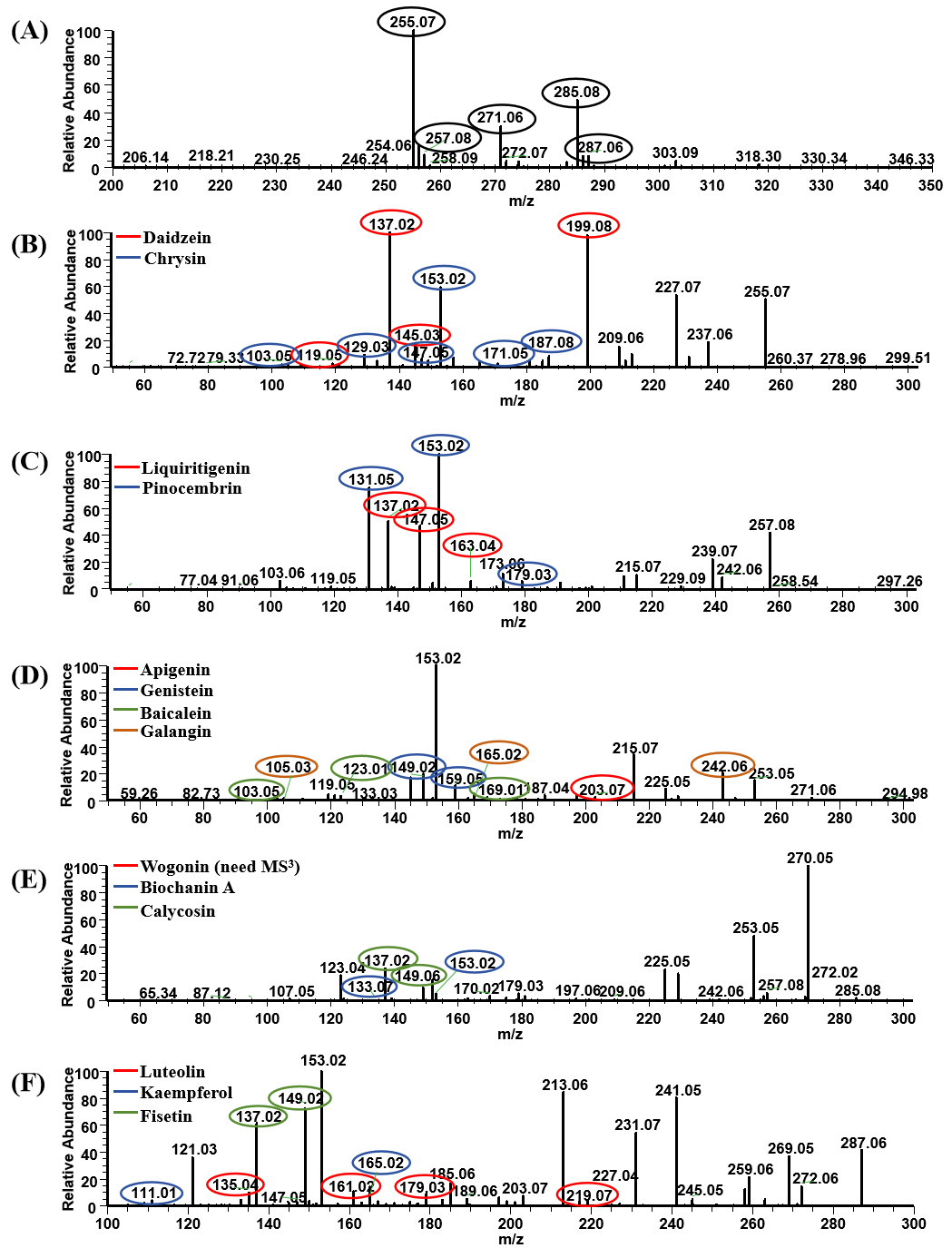


**Figure S8.** DART-MS^2^ analysis of mixture of five groups of flavonoid isomers including daidzein and chrysin (MW 254.06), liquiritigenin and pinocembrin (MW 256.07), apigenin, genistein, baicalein and galangin (MW 270.05), wogonin, biochanin A, and calycosin (MW 284.07), luteolin, kaempferol and fisetin (MW 286.05). (A) DART-MS analysis of flavonoid isomer mixtures, DART-MS^2^ analysis for molecular ion of (B) *m/z* 255.07, (C) *m/z* 257.08, (D) *m/z* 271.06, (E) *m/z* 285.08, and (F) *m/z* 287.06, respectively. MW indicates molecular weight.


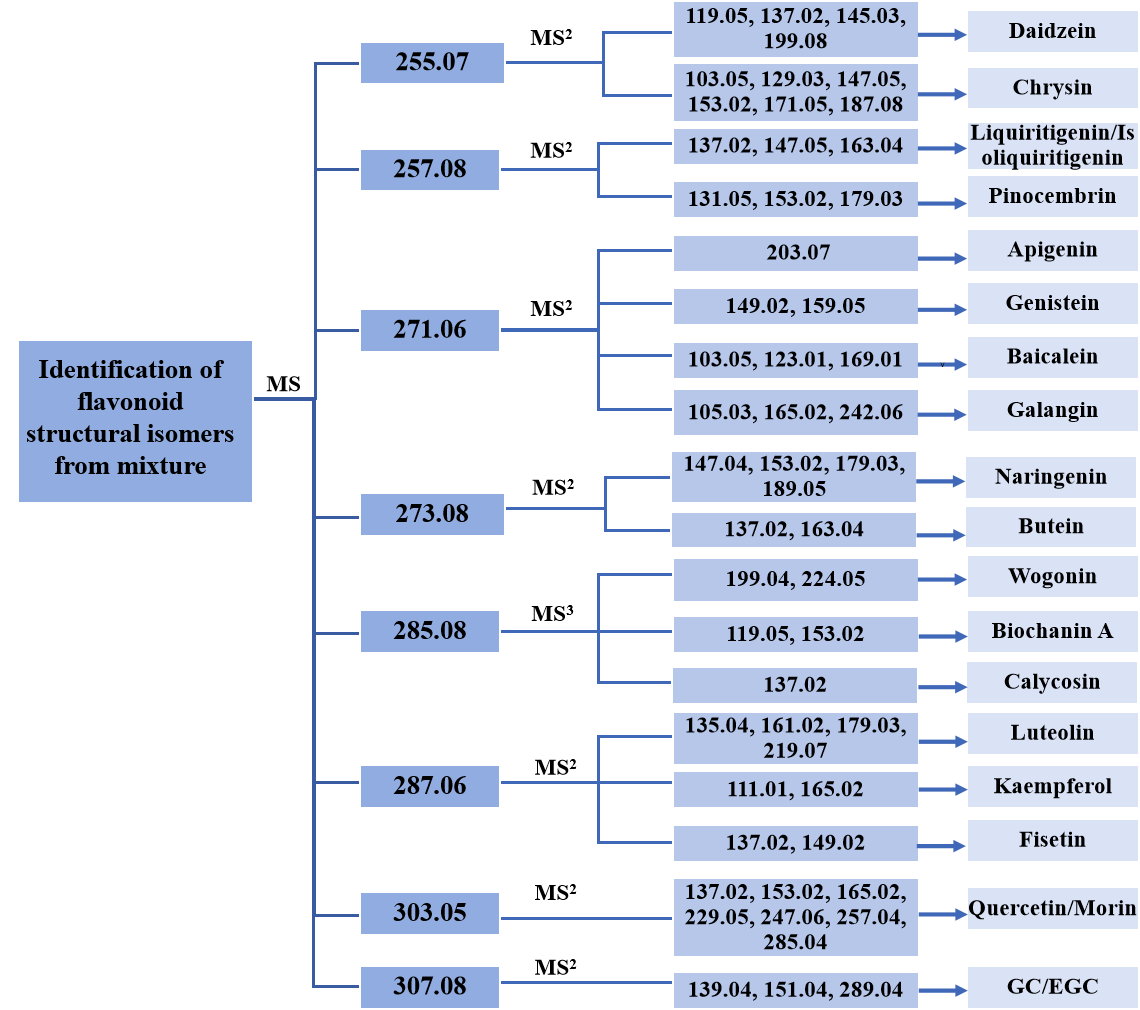


**Figure S9.** Schematic diagram of identification of flavonoid structural isomer from mixture using specific product ions obtained for each compound from DART-MS^n^ analysis.


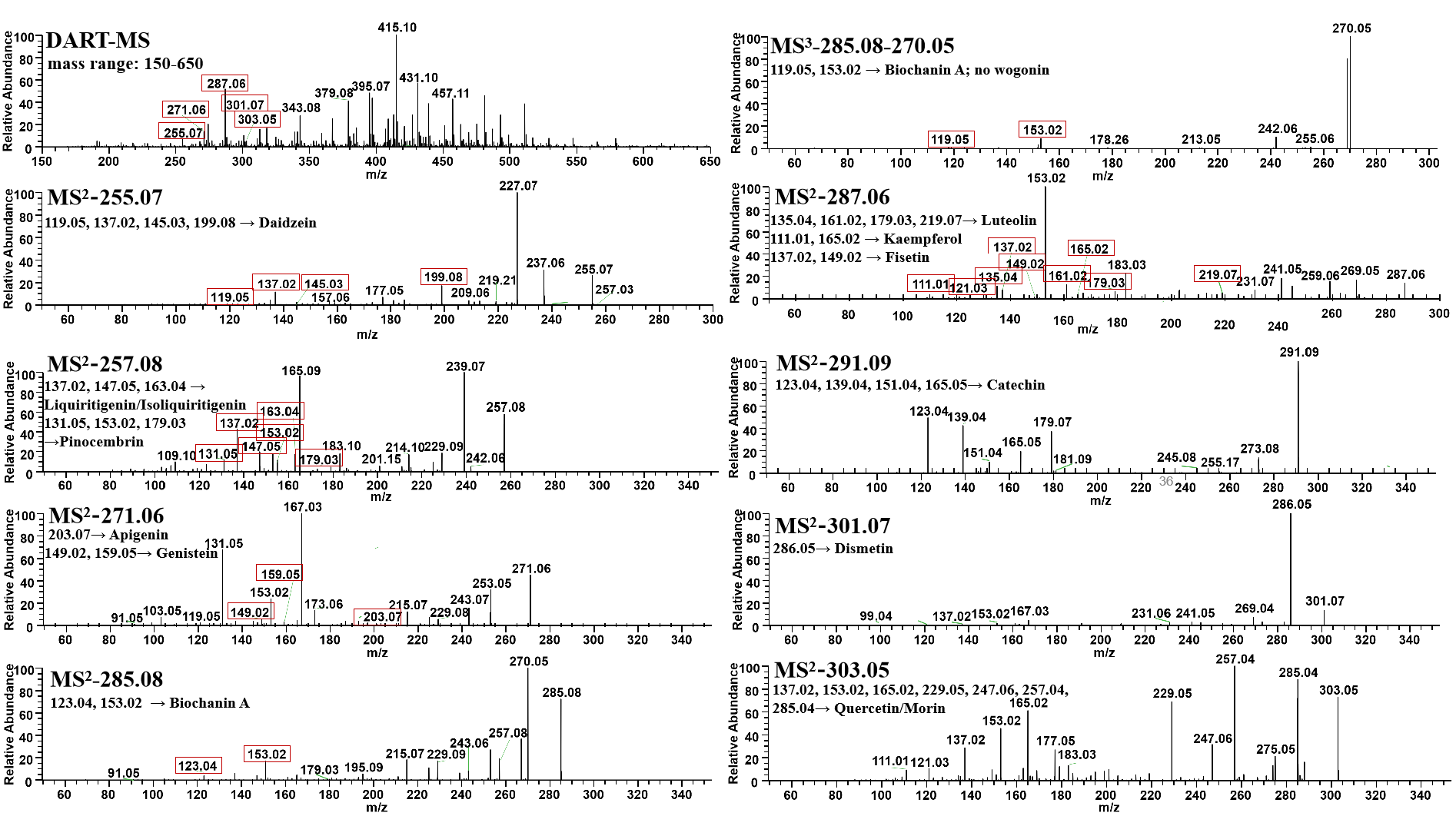


**Figure S10.** Full mass scan and DART- DART-MS^n^ analysis of cajan leaf extract. The identifications of different flavonoid compound are indicated on the spectra.


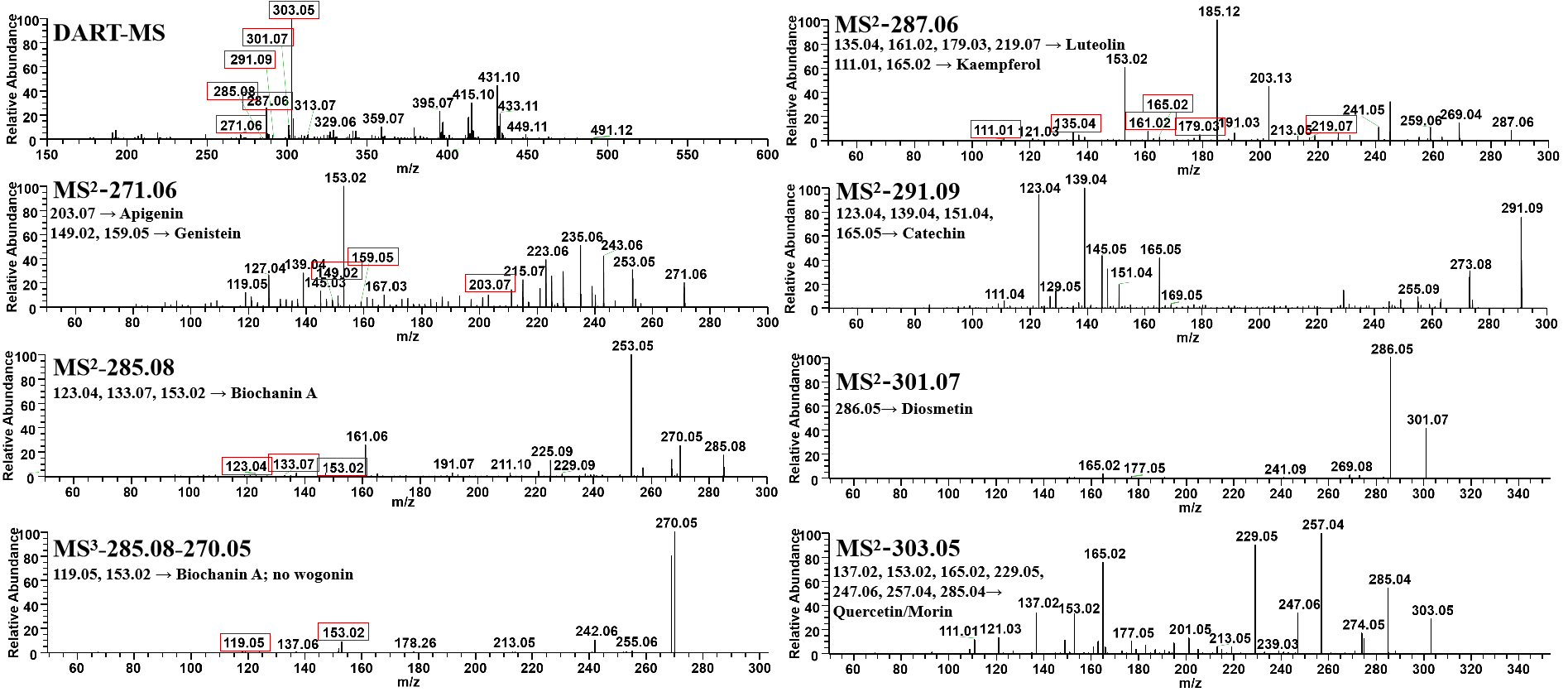


**Figure S11.** Full mass scan and DART- DART-MS^n^ analysis of rooibos tea extract. The identifications of different flavonoid compound are indicated on the spectra.


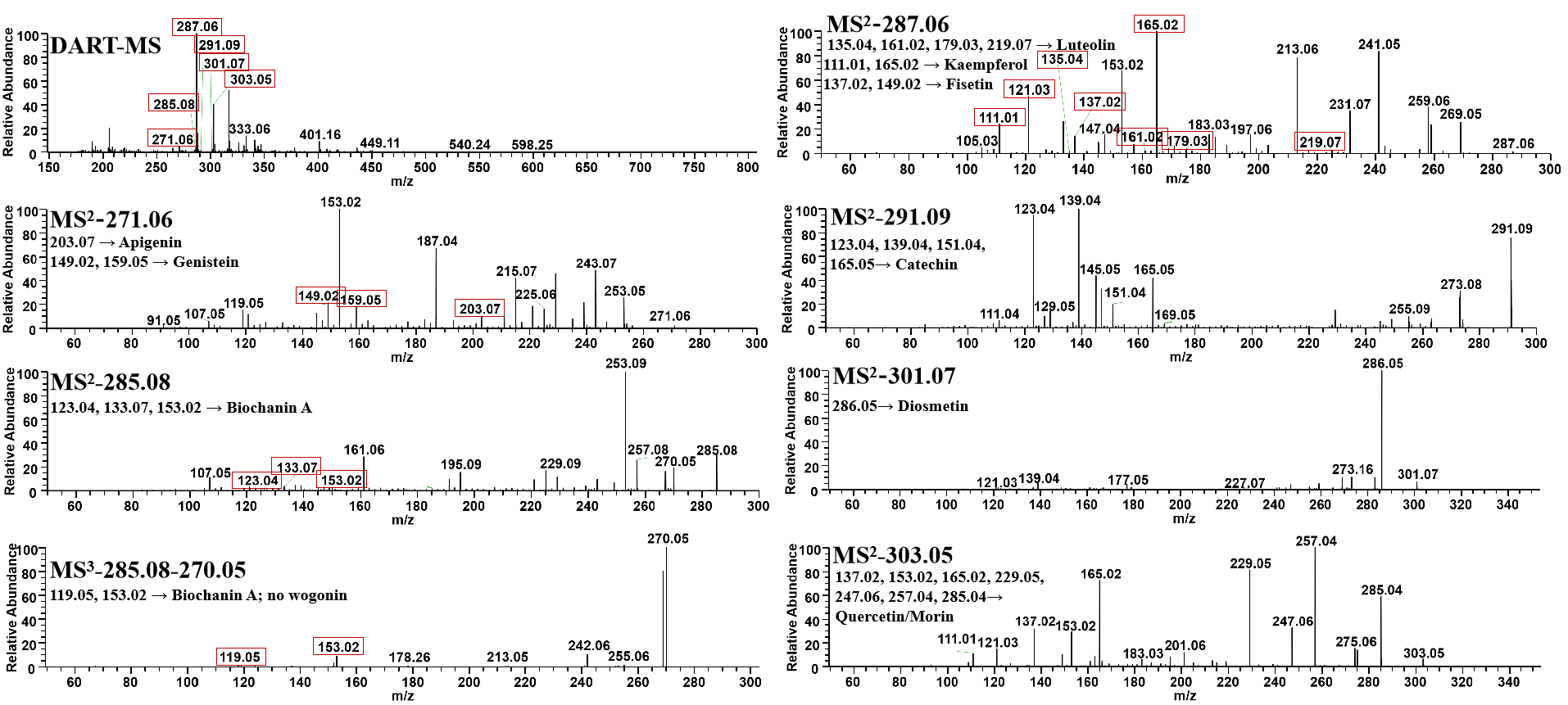


**Figure S12.** Full mass scan and DART- DART-MS^n^ analysis of ginkgo leaf extract. The identifications of different flavonoid compound are indicated on the spectra.


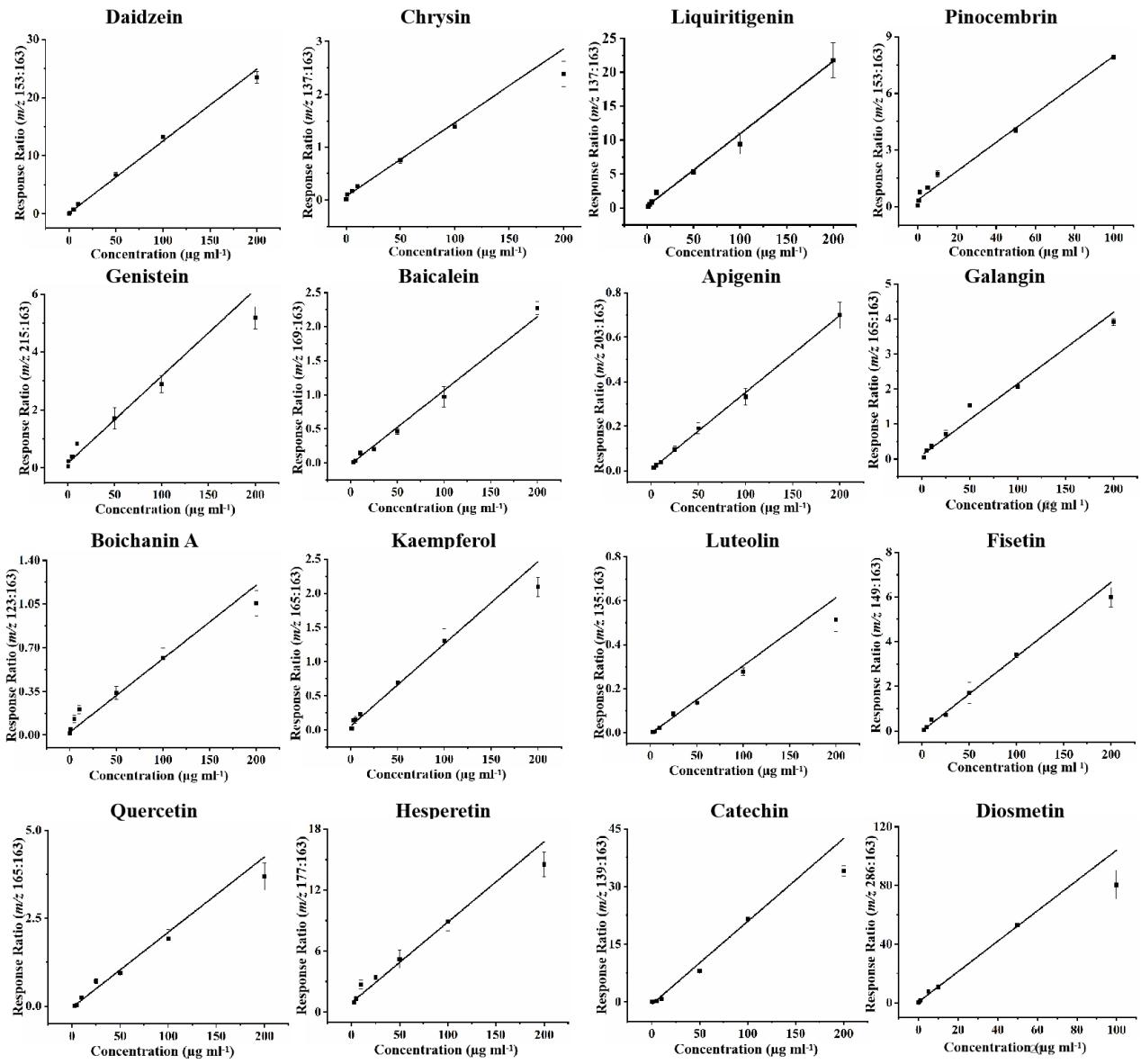


**Figure S13.** Calibration curves of 16 flavonoid compounds using DART-MS^2^ analysis. A series concentrations of standard solutions were used as x-axis, and the extracted peak area ratios of diagnostic product ion from flavonoid and fragment of IS (*m/z* 163) were used as y axis.

**Table S1.** List of flavonoid standards in this work with structural characteristics.


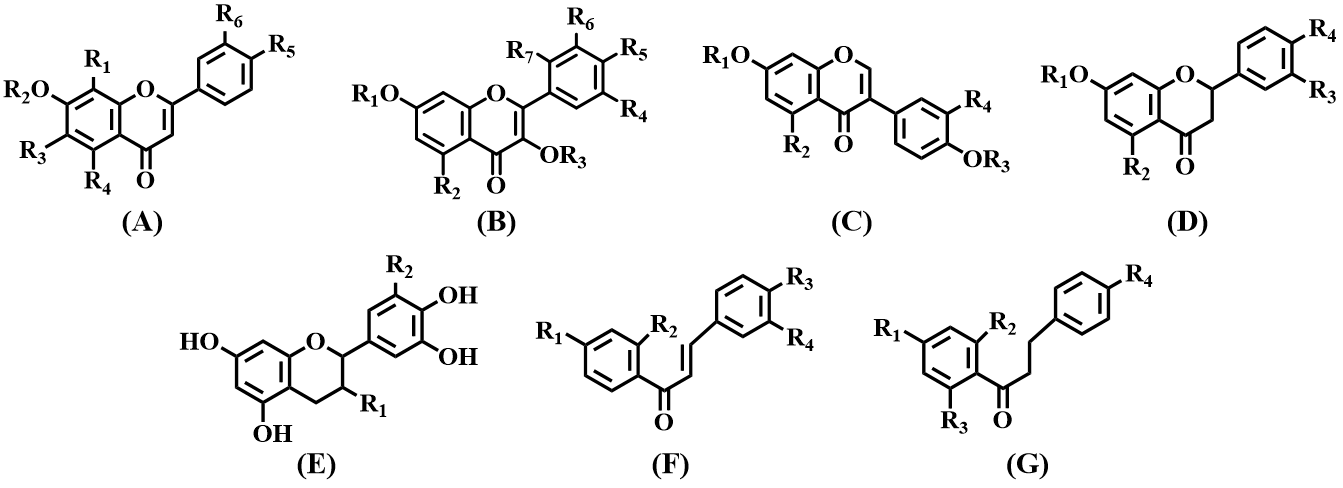


| **Flavonoid** | **M.W.(Da)** | **R_1_** | **R_2_** | **R_3_** | **R_4_** | **R_5_** | **R_6_** | **R_7_** | **Class** |
| --- | --- | --- | --- | --- | --- | --- | --- | --- | --- |
| Chrysin | 254.0579 | H | H | H | OH | H | H | -- | (A)  Flavone |
| Apigenin | 270.0528 | H | H | H | OH | OH | H | -- |  |
| Baicalein | 270.0528 | H | H | OH | OH | H | H | -- |  |
| Wogonin | 284.0685 | OCH_3_ | H | H | OH | H | H | -- |  |
| Luteolin | 286.0477 | H | H | H | OH | OH | OH | -- |  |
| Diosmetin | 300.0634 | H | H | H | OH | OCH_3_ | OH | -- |  |
| Galangin | 270.0528 | H | OH | H | H | H | H | H | (B)  Flavonol |
| Kaempferol | 286.0477 | H | OH | H | H | OH | H | H |  |
| Fisetin | 286.0477 | H | H | H | OH | OH | H | H |  |
| Quercetin | 302.0426 | H | OH | H | H | OH | OH | H |  |
| Morin | 302.0426 | H | OH | H | H | OH | H | OH |  |
| Myricetin | 318.0376 | H | OH | H | OH | OH | OH | H |  |
| Daidzein | 254.0579 | H | H | H | H | -- | -- | -- | (C)  Isoflavone |
| Genistein | 270.0528 | H | OH | H | H | -- | -- | -- |  |
| Biochanin A | 284.0685 | H | OH | CH_3_ | H | -- | -- | -- |  |
| Calycosin | 284.0685 | H | H | CH_3_ | OH | -- | -- | -- |  |
| Liquiritigenin | 256.0736 | H | H | H | OH | -- | -- | -- | (D)  Dihydroflavone |
| Pinocembrin | 256.0736 | H | OH | H | H | -- | -- | -- |  |
| Naringenin | 272.0685 | H | OH | H | OH | -- | -- | -- |  |
| Hesperetin | 302.0790 | H | OH | OH | OCH_3_ | -- | -- | -- |  |
| Catechin (C) | 290.0790 | H | H | -- | -- | -- | -- | -- | (E)  Flavane |
| Gallocatechin (GC) | 306.0739 | H | OH | -- | -- | -- | -- | -- |  |
| Epigallocatechin (EGC) | 306.0739 | H | OH | -- | -- | -- | -- | -- |  |
| 4-Methoxychalcone | 238.0994 | H | H | OCH_3_ | H | -- | -- | -- | (F)  Chalcone |
| Isoliquiritigenin | 256.0735 | OH | OH | OH | H | -- | -- | -- |  |
| Butein | 272.0685 | OH | OH | OH | OH | -- | -- | -- |  |
| Phloretin | 274.0841 | OH | OH | OH | OH | -- | -- | -- | (G)  Dihydrochalcone |

**Table S2.** The linear correlation coefficients, LODs, LOQs and RSDs for the 16 flavonoids.

| **Flavonoid** | **Calibration** | **R^2^** | **Reproducibility**  **RSD (%)** | **LOD (ng/ml)** | **LOQ (ng/ml)** |
| --- | --- | --- | --- | --- | --- |
| Chrysin | Y=0.01394x+0.06928 | 0.9968 | 8.31 | 9.4 | 31.4 |
| Daidzein | Y=0.12388x+0.08424 | 0.9980 | 9.65 | 0.2 | 0.7 |
| Liquiritigenin | Y=0.10680x+0.25441 | 0.9959 | 11.13 | 7.5 | 24.9 |
| Pinocembrin | Y=0.07621x+0.34871 | 0.9942 | 7.89 | 9.9 | 33.0 |
| Genistein | Y=0.02992x+0.16647 | 0.9951 | 7.99 | 37.0 | 123.3 |
| Baicalein | Y=0.01082x-0.01841 | 0.9964 | 10.02 | 0.2 | 0.7 |
| Apigenin | Y=0.00346x+0.00602 | 0.9993 | 5.19 | 328.1 | 1093.6 |
| Galangin | Y=0.02041x+0.10548 | 0.9914 | 9.56 | 0.5 | 1.7 |
| Biochanin A | Y=0.00588x+0.02411 | 0.9912 | 6.67 | 24.7 | 82.4 |
| Kaempferol | Y=0.0103x+0.12118 | 0.9934 | 7.89 | 2.4 | 8.1 |
| Luteolin | Y=0.00310x-0.00617 | 0.9979 | 11.72 | 524.6 | 1748.6 |
| Fisetin | Y=0.03330x-0.00768 | 0.9967 | 5.52 | 40.2 | 133.9 |
| Quercetin | Y=0.02142x-0.04353 | 0.9969 | 9.08 | 7.9 | 26.5 |
| Hesperetin | Y=0.07906x+0.93165 | 0.9935 | 6.35 | 285.3 | 951.0 |
| Catechin | Y= 0.21551x-0.62956 | 0.9914 | 16.30 | 0.9 | 3.1 |
| Diosmetin | Y= 1.03197x+0.64883 | 0.9902 | 7.74 | 0.5 | 1.8 |
